# Sustainable Electrochemical Strategy for Selective Double Bond Oxidation in the Presence of Aldehyde Groups with Mo─N─O Catalysts

**DOI:** 10.1002/advs.202506584

**Published:** 2025-06-23

**Authors:** Zhirong Li, Xun Zhang, Jiao Liu, Zhaomin Hao, Tengfei Zhang, Yang Yang, Yitong Wang, Santhosh Kamaraj, Jianrong Zeng, Qingsong Dong, Cuiping Zhai, Wuping Liao, Shuyan Song

**Affiliations:** ^1^ College of Chemistry and Molecular Sciences Henan University Kaifeng 475001 P. R. China; ^2^ Ganjiang Innovation Academy Chinese Academy of Sciences Ganzhou 341119 P. R. China; ^3^ Shanghai Synchrotron Radiation Facility Shanghai Advanced Research Institute Chinese Academy of Sciences Shanghai 201210 P. R. China; ^4^ Changchun Institute of Applied Chemistry Chinese Academy of Sciences Changchun 130022 P. R. China

**Keywords:** electrochemical, hydroperoxyl radicals, mechanistic studies, molybdenum‐based catalyst, selective oxidation

## Abstract

Selective oxidation of double bonds in aldehyde‐containing molecules is critical for synthesizing multifunctional organic compounds; however, conventional methods struggle to preserve aldehyde integrity due to competitive over‐oxidation. Here, an electrochemical strategy is presented that achieves precise double bond oxidation without aldehyde degradation, using hydroperoxyl radicals (^*^OOH) generated by a molybdenum‐based catalyst (Mo─N─O). Under mild, energy‐efficient conditions (−200 mA), this approach converts cinnamaldehyde to benzaldehyde with complete conversion (100%) and >99% selectivity, outperforming traditional harsh oxidants. Mechanistic analysis reveals that the tailored coordination environment of the catalyst enhances ^*^OOH formation, enabling targeted oxidative activity. The method operates at ambient conditions, eliminates toxic reagents and high energy‐consuming process, and aligns with sustainable chemistry principles. By integrating electrocatalysis with insights from density functional theory, the study establishes a scalable platform for the precise transformation of multifunctional organic molecules, representing a significant step forward in energy‐efficient chemical synthesis.

## Graphical abstract

In this work, an electrochemical strategy employing hydroperoxyl radicals and a molybdenum‐based catalyst (Mo─N─O) enables selective double‐bond oxidation in cinnamaldehyde, preserving aldehyde integrity. Under mild, energy‐efficient conditions (−200 mA), this approach converts cinnamaldehyde to benzaldehyde with complete conversion (100%) and >99% selectivity, outperforming traditional harsh oxidants.

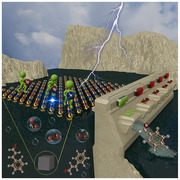

## 1 Introduction

Recent advances in sustainable synthesis underscore the significance of elucidating the interactions for different functional groups during oxidation reactions.^[^ ^1^ ^]^ In particular, organic molecules that contain both aldehyde groups and double bonds offer a compelling challenge due to their contrasting reactivity.^[^ ^1c–f^ ^]^ Addressing these challenges is pivotal for advancing sustainable synthesis, particularly in designing energy‐efficient processes that align with green chemistry principles.^[^ ^1^, ^2^ ^]^ Generally, the aldehyde group exhibits higher reactivity toward oxidation compared to double bonds. Under mild oxidative conditions, the aldehyde group is frequently converted to a carboxylic acid, while the double bonds generally remain unaltered due to the selective nature of oxidizing agents such as Fehling's solution and Tollens reagent.^[^ ^3^ ^]^ In contrast, under more robust oxidative conditions employing agents like potassium permanganate (KMnO_4_) or ozone (O_3_), both the aldehyde group and the double bonds may undergo transformation.^[^ ^3^, ^4^ ^]^ Initial investigations into these reactions revealed that double bonds might cleave under specific oxidative conditions, forming short‐chain carboxylic acids or ketones.^[^ ^5^ ^]^ The exact transformation pathway is influenced by the compound's structure and the oxidizing agent applied. As a result, oxidation products range from selective aldehyde oxidation to comprehensive modifications involving both functional groups.^[^ ^3^, ^4^, ^6^ ^]^

Understanding the applicability and limitations of oxidative agents in such transformations is essential due to their theoretical and practical significance in synthetic organic chemistry.^[^ ^3^, ^4^, ^6^, ^7^ ^]^ Unfortunately, traditional approaches for achieving selective oxidation of double bonds in the presence of aldehyde groups often require harsh reaction conditions, including elevated temperature and pressure, as well as stringent anaerobic and anhydrous environments (Figure S1, Supporting Information).^[^ ^3^, ^6^, ^8^ ^]^ Such challenges have significantly hindered their broader application in organic synthesis. Therefore, developing milder, more selective, and environmentally friendly oxidative processes for double bonds in the presence of aldehyde groups remains a key objective.^[^ ^1^, ^3^, ^4^, ^6^ ^]^ Such advancements would improve the efficiency and specificity of synthetic pathways, aligning with the increasing demand for greener and more sustainable chemical practices. Additionally, addressing these challenges would expand the utility of selective oxidation techniques, enabling the synthesis of complex organic molecules and advancing the field of organic chemistry.^[^ ^4^, ^6^, ^7^ ^]^

Significant efforts have been devoted to mild conditions to achieve the oxidative reaction of double bonds in the presence of aldehyde groups.^[^ ^3^, ^9^ ^]^ However, most current reactions result in a diverse array of products, contribute to environmental unsustainability, and require complex catalysts.^[^ ^1^, ^6^, ^8^, ^9^ ^]^ Electrochemical oxidation, which uses electrical energy for chemical transformations and eliminates the need for high temperatures, pressures, and hazardous agents, has gained significant attention for its scalability and ability to generate reactive oxygen species like ^*^OH and ^*^OOH radicals at the electrode surface, enhancing the selectivity and efficiency of oxidation reactions.^[^ ^7^, ^10^ ^]^ Nevertheless, this approach is seldom accomplished when extended to achieve selective transformations of double bonds in the presence of aldehyde groups. Recent advances have demonstrated the selective oxidation of olefinic double bonds to alcohols via electrochemical methods, but extending this approach to systems containing both double bonds and active aldehyde groups poses significant challenges.^[^ ^15^ ^]^ The concurrent presence of these functional groups complicates the reaction pathways, often leading to undesired byproducts.

In this study, we propose an electrochemical strategy for the selective oxidation of double bonds in the presence of aldehyde groups, exemplified by the transformation of cinnamaldehyde into benzaldehyde. Using Mo─N─O catalysts, reactive ^*^OOH species are generated to enable selective oxidation under mild conditions, achieving high product selectivity while adhering to principles of environmental sustainability (Figures S2 and S3, Supporting Information). Density functional theory (DFT) calculations elucidate that modifications in the coordination environment around the metal active sites in Mo─N─O catalysts enhance ^*^OOH formation, thereby improving oxidative activity. This mechanistic insight highlights the potential of electrochemical approaches to overcome the challenges associated with the selective oxidation of complex organic molecules, providing a scalable and environmentally friendly alternative to traditional methods.

## 2 Results and Discussion

**Figure 1:**
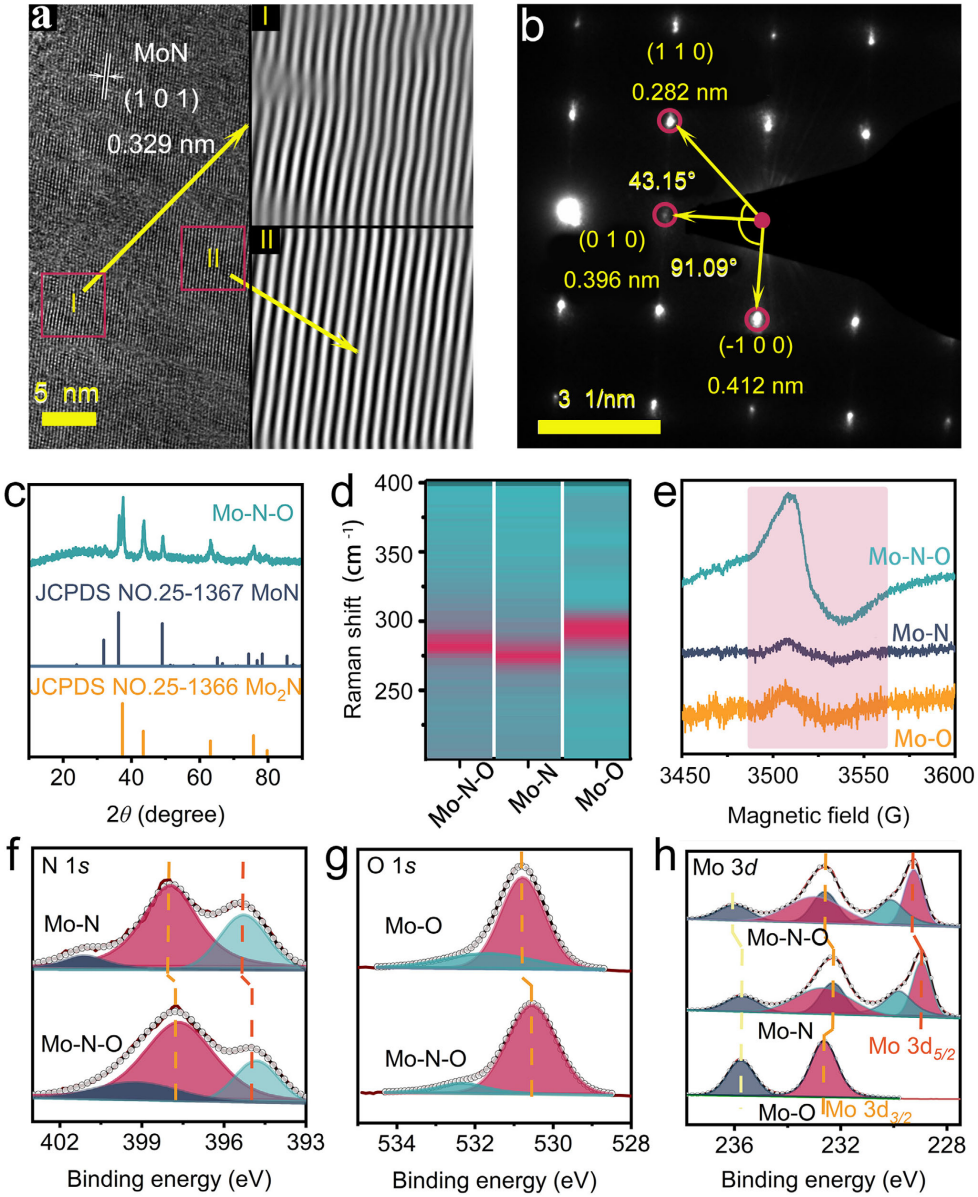
Characterization of Mo─N─O: a) HR‐TEM image; b) SAED pattern; Comparison of c) XRD, d) Raman and e) EPR signals for Mo─N─O and its control samples; Comparison of f) Mo 3*d*, g) N1*s* and h) O1*s* XPS signals for Mo─N─O and its control samples.

Herein, we employed a new nitrogen/oxygen co‐doped molybdenum‐based material (denoted as Mo─N─O) as a catalyst, mainly inspired by our previous findings that transition‐metal oxynitrides exhibit superior performance compared to their nitride or oxide counterparts in two‐electron oxygen reduction reactions (2*e* ^−^ ORR).^[^ ^11^ ^]^ Generally, the Mo─N─O catalyst was synthesized using a two‐step solid‐phase method under high‐temperature conditions (see Methods in Supporting Information). To elucidate the composition and surface characteristics of the Mo─N─O catalyst and to establish correlations between structure and performance, comprehensive characterizations were conducted. The results from scanning electron microscopy (SEM) indicate that the surface of Mo─N─O has become markedly rough and formed a porous structure due to high‐temperature treatment (Figures S4–S6, Supporting Information). High resolution transmission electron microscopy (HR‐TEM) images revealed the presence of the MoN (101) crystal planes on the catalyst's surface, with an interplanar distance of 0.329 nm. Fourier transform and inverse Fourier transform analyses of the HR‐TEM images uncovered two distinct types of lattice stripes within this crystal plane (**Figure** **1**a). Lattice stripes in Region II exhibited orderly and straight arrangements, while those in Region I displayed pronounced distortion, indicating different lattice characteristics from Region I. This phenomenon is attributed to the introduction of heteroatoms, causing initial lattice distortion, which is consistent with previous literature.^[^ ^12^ ^]^ Selected area electron diffraction (SAED) further confirmed that the Mo─N─O catalyst is indeed a nitrogen/oxygen co‐doped catalyst. The angles of crystal planes between (010) and (‐100), as well as (110) and (010), were 91.09° and 43.15°, deviating from the ideal cubic angles of 90° and 45° (Figure 1b).

Powder X‐ray diffraction (XRD) patterns demonstrated that the primary chemical constituents of Mo─N─O are MoN (JCPDS No. 25–1367) and Mo_2_N (JCPDS No. 25–1366) (Figure 1c). A comparative XRD analysis of Mo─N─O and its nitride precursor after the first annealing step (denoted as Mo─N) showed highly similar diffraction peaks, distinct from those of the initial oxide precursor (denoted as Mo─O) (Figure S7, Supporting Information). Given the fact that XRD is a macroscopic characterization technique capable of penetrating the bulk of materials rather than just probing their surface layers, we speculate that the oxygen in the Mo─N─O catalyst may be present primarily as a surface‐doped element, without significantly altering the primary chemical composition. Energy‐dispersive X‐ray spectroscopy (EDS) elemental mapping images revealed that molybdenum, nitrogen and oxygen were uniformly distributed on the surface of the Mo─N─O catalyst (Figures S8–S10, Supporting Information). To further elucidate the differences between Mo─N─O and its precursors, Raman spectroscopy—a non‐destructive technique for studying material structure and chemical composition—was employed.^[^ ^13^ ^]^ Raman spectroscopy indicated characteristic peaks at ≈294.6, 274.9, and 281.9 cm^−1^ for Mo─O, Mo─N, and Mo─N─O, respectively (Figure 1d). Notably, the characteristic peak of Mo─N─O exhibited a redshift of 12.7 cm^−1^ compared to Mo─O, and a blueshift of 7 cm^−1^ compared to Mo─N. These measurements confirm the successful preparation of the Mo─N─O material with an N/O co‐doped surface using our synthesis protocol.

Electrons or holes trapped in or around the doping sites of heteroatoms can lead to the formation of significant vacancies in the material.^[^ ^12^, ^14^ ^]^ To investigate these vacancies on the surface of Mo─N─O, electron paramagnetic resonance (EPR) spectroscopy was conducted. As expected, compared to Mo─O and Mo─N, the EPR spectra and the increased signal intensity at the *g*‐factor (*g* = 1.919 ± 0.002) indicated a higher concentration of unsaturated Mo vacancies with unpaired electrons in Mo─N─O (Figure 1e; Figure S11, Supporting Information).^[^ ^15^ ^]^ To further evaluate the impact of N/O doping on Mo─N─O, X‐ray photoelectron spectroscopy (XPS) measurements were employed to compare the surface composition.^[^ ^16^ ^]^ The N 1*s* doublet is shifted by 0.3 eV (Figure 1f), and the O 1*s* singlet is shifted by 0.2 eV (Figure 1g) in Mo─N─O compared to their precursors. Furthermore, the Mo 3*d* characteristic peaks of Mo─O, Mo─N, and Mo─N─O exhibit distinct positional shifts (Figure 1h). Specifically, the Mo 3*d*3/2 peak of Mo─N─O at ≈232.6 eV is similar to that of Mo─O but shows a 0.3 eV shift compared to Mo─N. Another Mo 3*d*3/2 peak of Mo─N─O appears at 236 eV, which is blueshifted by 0.2 and 0.3 eV relative to Mo─O and Mo─N, respectively. Additionally, a characteristic Mo 3*d*5/2 peak at 229.3 eV in Mo─N─O is blueshifted by 0.2 eV compared to Mo‐N. These XPS results indicate that the co‐doping of N and O alters the chemical environment of the central metal and influences its electronic structure.

**Figure 2:**
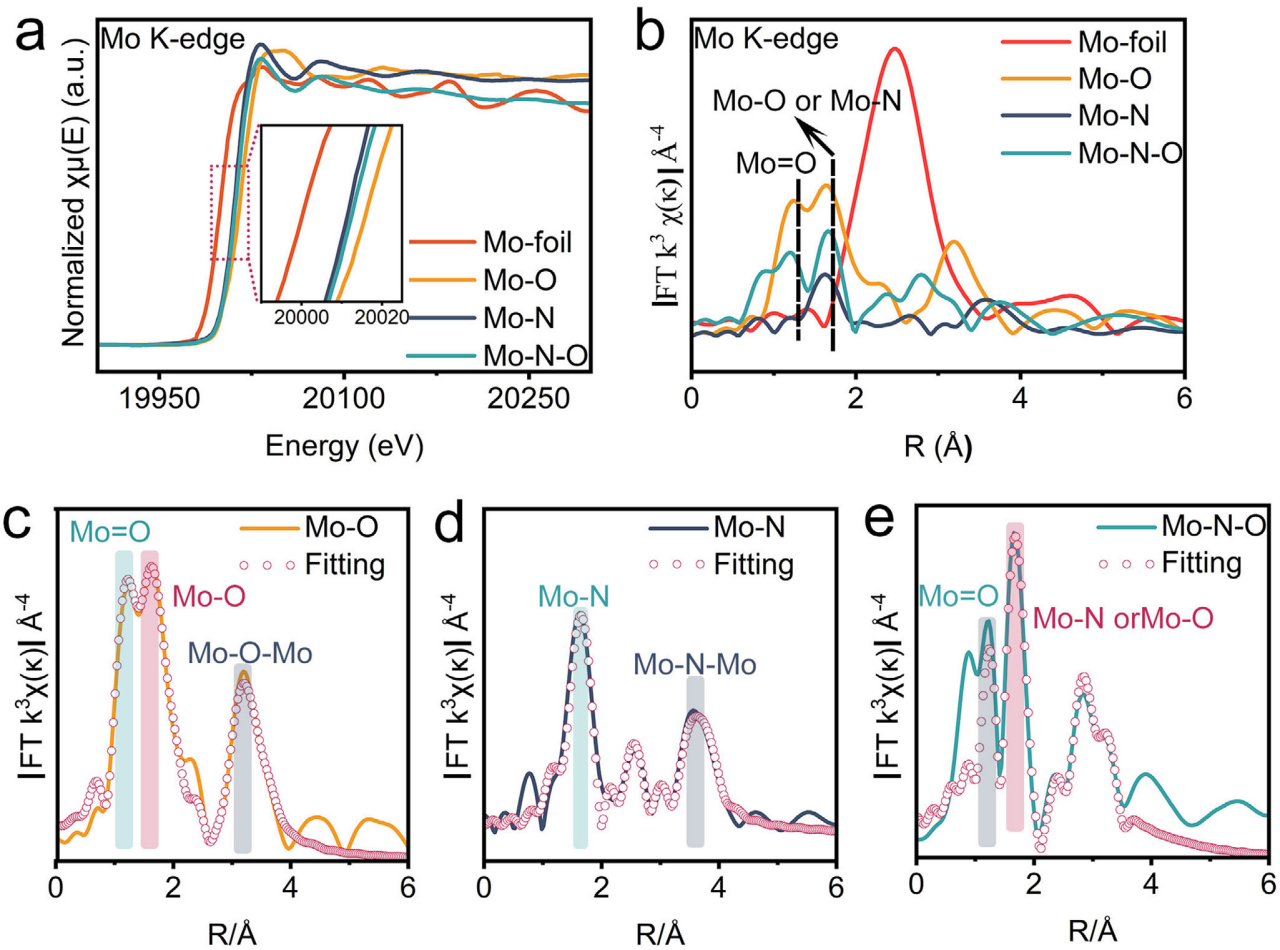
Chemical states and coordination structures characterized by XANES and EXAFS: a) Energy space of Mo foil, Mo─O, Mo─N and Mo─N─O at the Mo *K*‐edge; b) *R*‐space spectra of the Mo─O, Mo─N and Mo─N─O; Observed and fitted *R*‐space spectra of c) Mo─O, d) Mo─N and e) Mo─N─O.

Synchrotron radiation techniques, including X‐ray absorption near‐edge structure (XANES) and extended X‐ray absorption fine structure (EXAFS), could provide detailed insights into the coordination environment and electronic structure of molybdenum in Mo─N─O at the atomic level.^[^ ^17^ ^]^ The oxidation state of catalysts can typically be inferred from the position of the normalized χμ(E) curve. The positions of Mo─N─O and Mo‐N lie between those of Mo foil (0) and Mo─O (+6). The calculated results indicate that the valence state of the central atom (Mo^x+^) in Mo─N is +4.40, while the introduction of oxygen atoms in Mo─N─O slightly increases the valence state of Mo to +4.72 (**Figure** **2**a; Figure S12, Supporting Information). Additionally, the curve for Mo─N─O is closer to that of Mo─O, suggesting a slight increase in oxidation state following oxygen doping in the pure nitride, which aligns with XPS results. Fourier transform (FT) is a crucial tool for data extraction and interpretation of EXAFS spectra. Analysis of the Mo *K*‐edge FT‐EXAFS spectra reveals that the Mo─Mo bond length in the reference Mo‐foil is ≈2.5 Å (Figure 2b). The splitting of the Mo─O bond peak into two overlapping peaks (≈1.2 and 1.6 Å) indicates the presence of two distinct Mo─O bond lengths in Mo─O. For the precursor, the Mo─N bond length is closer to the longer Mo─O bonds, measuring ≈1.6 Å. Notably, the Mo─N─O exhibits a new peak at 1.2 Å, indicating shorter Mo═O bonds, which provides direct evidence for the successful synthesis of N/O co‐doped Mo‐based catalysts.^[^ ^18^ ^]^ Furthermore, the ratio of peak intensities at *R* = 1.2 Å and *R* = 1.6 Å for Mo─O and Mo─N─O is 1:1.04 and 1:1.25. This suggests that the peaks corresponding to Mo─N bonds overlap with those of Mo─O bonds, resulting in changes in the peak intensity ratio and confirming the presence of two different Mo─O bond lengths in the Mo─N─O electrocatalysts.

We subsequently performed quantitative fitting of the scattering paths in the EXAFS results, focusing on a fitting range of *R* = 1–4 Å. The optimal fitting results for the first and second scattering shells in the *R*‐space of Mo─O, Mo─N, and Mo─N─O are illustrated in Figure 2c–e, respectively. Additionally, the *K*‐space data and their corresponding fitting results are provided in Figures S13–S16 (Supporting Information). Wavelet transform (WT) analysis offers a high‐resolution and intuitive representation of the coordination environment.^[^ ^19^ ^]^ Specifically, the bright spot observed at *K* = 10.9 Å^−1^ corresponds to the first shell path of the Mo─O bond at *R* = 1.5 Å, while the bright spot at *K* = 13.8 Å^−1^ and *R* = 3.8 Å represents the second shell X‐ray scattering path of Mo─O─Mo (Figure S18, Supporting Information). As depicted in Figure S19 (Supporting Information), the strongest intensity spans a broad range due to the overlap between the first shell (Mo─N bond, *K* = 13.2 Å^−1^ and *R* = 1.6 Å) and the second shell (Mo─N─Mo, *K* = 13.9 Å^−1^ and *R* = 3.0 Å). Similarly, the Mo─N─O shows a peak at *K* = 12.2 Å^−1^ and *R* = 1.3 Å, indicating overlapping signals from Mo─O and Mo─N bonds. This peak also represents the combined signals of the Mo─N─Mo and Mo─O─Mo scattering paths. Furthermore, compared with the wavelet transformation of Mo‐foil, the Mo─N─O does not exhibit a Mo─Mo coordination pattern, corroborating the conclusions drawn from the *R*‐space comparison (Figure 2b; Figures S17 and S20, Supporting Information).

**Figure 3:**
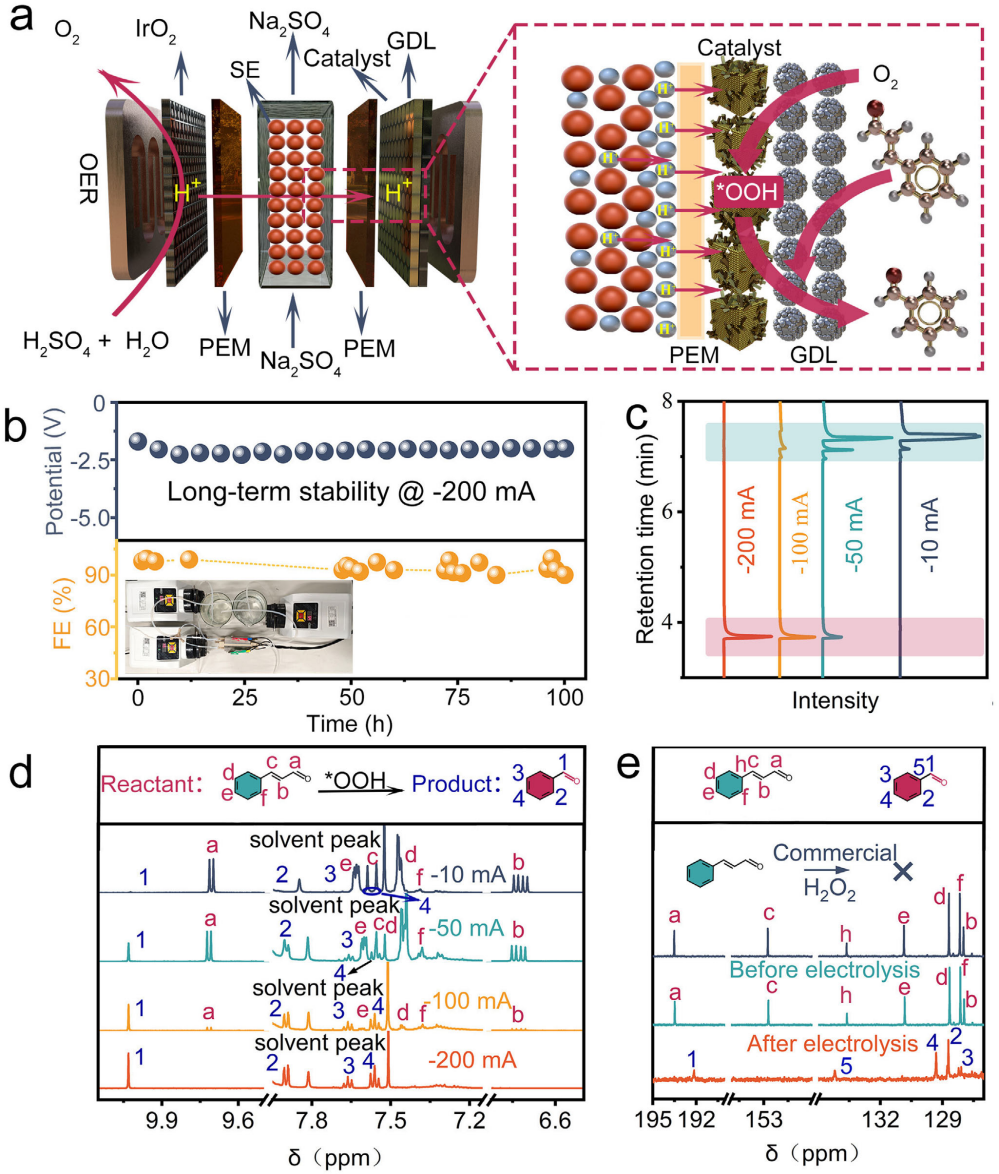
Evaluation of the electrochemical performance: a) The two‐PEM electrolytic cell used in the experiment; b) The curve for the long‐term stability of the Mo─N─O catalyst (left corner, reaction setup); c) Signals of gas chromatography under various currents; d) The ^1^HNMR spectrum of products under various currents; e) Comparison of ^13^CNMR spectrum (black, using commercial H_2_O_2_; cyan, before electrolysis; orange, after electrolysis).

The unique structural features of Mo─N─O present significant opportunities as catalysts in a range of intriguing chemical reactions. This study investigates the use of Mo─N─O to achieve the selective oxidation of cinnamaldehyde under mild conditions—a reaction of particular interest due to its industrial relevance and potential for fine chemical synthesis. The experimental process was primarily conducted in an electrolytic cell where oxygen (O_2_) diffused through a gas diffusion layer (GDL) to reach the catalyst surface, initiating the ORR, as illustrated in **Figure** **3**a. During the ORR, two primary pathways are possible: the 2‐electron (2*e*⁻) process, which leads to the production of hydroperoxide (^*^OOH), and the 4‐electron (4*e*⁻) process, resulting in the formation of ^*^O, ^*^OH, and ^*^OOH intermediates.^[^ ^20^ ^]^ To determine the predominant ORR pathway facilitated by Mo─N─O in the selective oxidation of organic molecules, we utilized a rotating ring‐disk electrode (RRDE) setup. The RRDE experiments revealed that the electron transfer number for Mo─N─O was ≈2.06, with a 2*e* ^−^‐ORR selectivity of 96.6% (Figures S23 and S24, Supporting Information). These results indicate that Mo─N─O primarily follows the 2*e*⁻ pathway, generating ^*^OOH radicals during the ORR.

In the electrolytic cell, cinnamaldehyde was introduced as a substrate to explore its transformation through in situ generated ^*^OOH radicals. These radicals reacted with cinnamaldehyde, promoting the conversion of this organic molecule under ambient conditions. The magnitude of the applied current significantly influences the yield of ^*^OOH radicals, thereby affecting the diversity and yield of reaction products. To optimize the conditions for the oxidation of cinnamaldehyde, we performed experiments at various currents: −10, −50, −100, −200, and −300 mA. The results indicated that the Faradaic efficiency (FE) initially increased with the applied current, reaching a maximum value of 94.9% at −200 mA before decreasing (Figure S25, Supporting Information). To further clarify the role of the N─O co‐coordination environment, we compared the catalytic performance of Mo─N─O with that of Mo─O and Mo─N, as well as some reported catalysts. At −200 mA, Mo─N─O exhibited a significantly higher FE (94.89%) than Mo‐O (63.77%) and Mo‐N (36.34%) and outperformed previously reported catalysts such as Mo‐TiO_2_, α‐MoO_3‐x_, Pr_2_Ni_0.8_Mo_0.2_O_4+δ_ (Figures S26 and S27, Supporting Information). These results underscore the critical contribution of nitrogen and oxygen coordination in facilitating ^*^OOH radical formation, thereby enhancing both the activity and selectivity. To evaluate the electrochemical stability and durability of the Mo─N─O catalyst in generating ^*^OOH radicals, a 100‐h continuous electrolysis experiment was performed at a current of −200 mA. The curves of Faradaic efficiency (FE) and bias, alongside the comparison of XRD spectra before and after the 2*e* ^−^ ORR, highlight the catalyst's robustness under prolonged operational conditions (Figure 3b; Figure S28, Supporting Information).

Building upon the superior ^*^OOH generation capability of Mo─N─O, we carried out additional electrolysis experiments on cinnamaldehyde at currents of −10, −50, −100, and −200 mA. Post‐electrolysis, the resultant organic electrolyte was processed via extraction and subjected to comprehensive analysis using gas chromatography (GC) and proton nuclear magnetic resonance (^1^HNMR) spectroscopy (Figure 3c,d). The retention times for benzaldehyde and cinnamaldehyde on GC were 3.7 and 7.4 min, respectively. Both GC and ^1^HNMR analyses indicated a gradual decrease in cinnamaldehyde concentration with a concurrent increase in benzaldehyde concentration. Detailed peak analysis via ^1^HNMR showed that only the carbon–carbon (C═C) double bonds were cleaved during the reaction, leading to the selective formation of benzaldehyde without oxidation of the aldehyde group. Importantly, no by‐products such as cinnamic acid, benzoic acid, or benzyl alcohol were detected, as evidenced by Figure 3d. This result highlights the exceptional selectivity of the Mo─N─O‐mediated process. To further elucidate the mechanism underlying ^*^OOH‐mediated selective oxidation, we examined the reactivity of four representative substrates—4‐pentenal (an allylic/β‐substituted enal), α‐methylstyrene ketone (an enone), cinnamic acid (an alkenyl acid), and citral (a structurally complex natural product). Their transformation profiles were systematically compared using NMR spectroscopy (Figures S29–S33 and Table S1, Supporting Information). These model studies yielded three key mechanistic insights. First, the 2*e* ^−^ ORR pathway preferentially targets enals with shorter carbon backbones. Second, substrate susceptibility is jointly governed by steric effects and the presence of the aldehyde functional group. Third, aromatic substitution stabilizes the aldehyde intermediate, thereby suppressing overoxidation. Together, these results underscore the substrate‐dependent behavior of ^*^OOH‐mediated oxidation and provide a mechanistic basis for the rational design of electrocatalytic systems with enhanced chemoselectivity.

To further validate the unique role of ^*^OOH in double bond oxidation, we performed ^13^C nuclear magnetic resonance (^13^CNMR) analysis on selected samples, including commercial H_2_O_2_, pre‐electrolysis cinnamaldehyde, and post‐electrolysis products at −200 mA. As shown in Figure 3e, the ^13^CNMR spectra for commercial H_2_O_2_ and pre‐electrolysis samples were identical except for the raw material cinnamaldehyde. However, after electrolysis, only the characteristic peak of benzaldehyde was observed. These results indicate that commercial H_2_O_2_ does not facilitate selective oxidation, whereas ^*^OOH is essential for achieving selective double‐bond oxidation in the presence of aldehyde groups. These findings conclusively demonstrate that the Mo─N─O catalyst effectively mediates the electrochemical oxidation of C═C double bonds in aldehyde‐containing compounds under mild conditions, without oxidizing the aldehyde or other functional groups.

**Figure 4:**
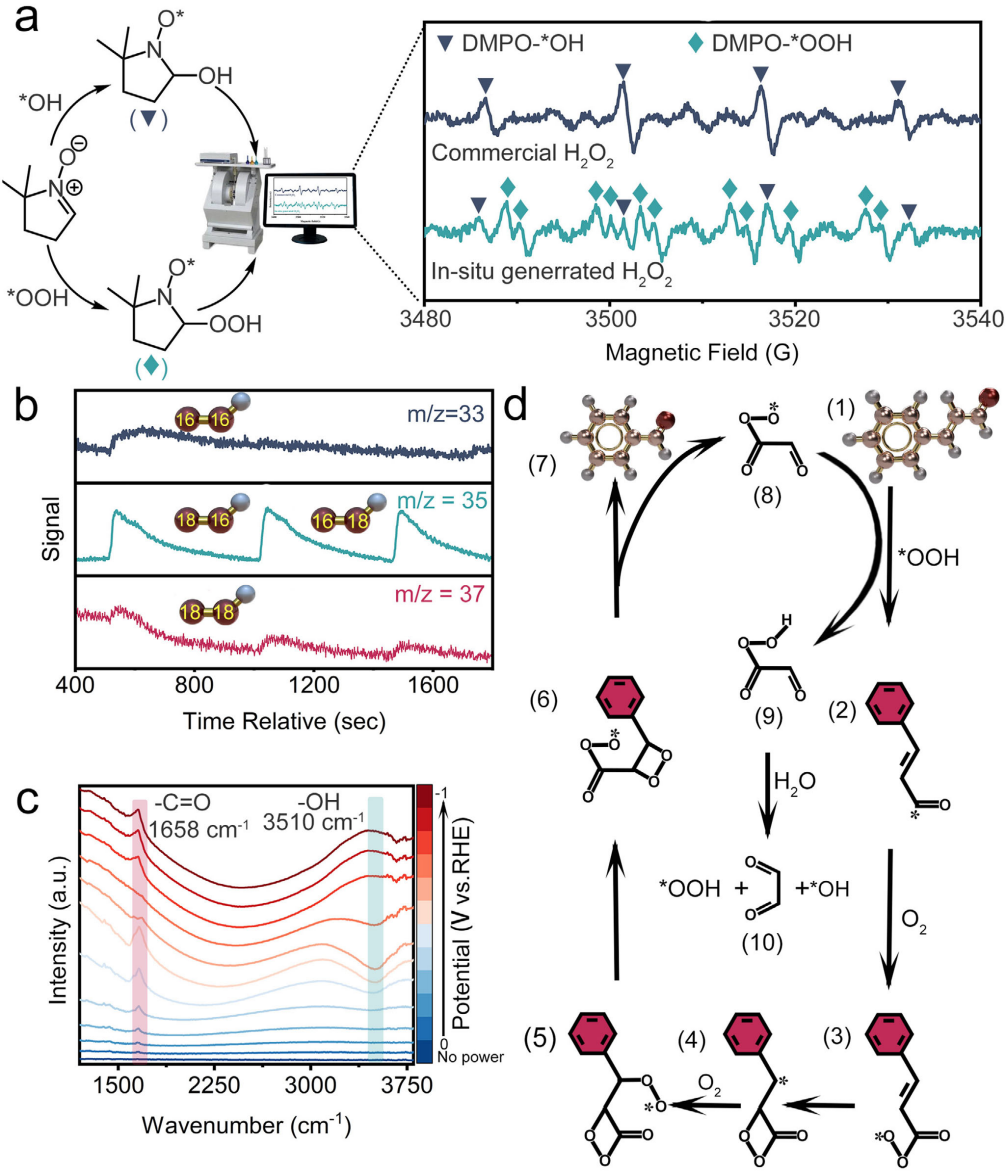
In situ experiments and proposed mechanism: a) EPR curves of DMPO‐^*^OOH and DMPO‐^*^OH from the in situ generated aqueous solution; b) Comparison of ^18^O and ^16^O‐labeled in situ DEMS signals; c) Comparison of *operando* ATR‐FTIR curves at various testing voltages; d) The proposed mechanism of conversion from cinnamaldehyde to benzaldehyde.

To elucidate the oxidative activity of double bonds in the presence of aldehydes, we conducted radical trapping experiments combined with electron paramagnetic resonance spectroscopy to detect radical signals. As shown in **Figure** **4**a, hydroxyl radicals (^*^OH) and hydroperoxyl radicals (^*^OOH) were effectively captured using 5,5‐dimethyl‐1‐pyrroline N‐oxide (DMPO), forming DMPO‐^*^OH and DMPO‐^*^OOH adducts, respectively. The EPR results revealed that in situ ORR could provide signals for both ^*^OH and ^*^OOH, whereas commercial H_2_O_2_ showed only ^*^OH signals. This strongly indicates that the oxidative activity in double‐bond oxidation is primarily attributed to ^*^OOH rather than ^*^OH. Further evidence for the generation of ^*^OOH and its role in oxidation reactions was obtained using in situ differential electrochemical mass spectrometry (DEMS) (Figure 4b). By labeling oxygen with the isotope ^18^O_2_, the mass spectrometer detected charged particle signals with mass‐to‐charge ratios (m/z) of 35 and 37, corresponding to ^*16^O^18^OH, ^*18^O^16^OH, and ^*18^O^18^OH, respectively. In contrast, signals for unlabeled particles (m/z = 33, representing ^*16^O^16^OH) were negligible, confirming that the Mo─N─O catalyst effectively converts O_2_ into ^*^OOH, which subsequently participates in the oxidation reaction.

To monitor changes in intermediates and reactants during the catalytic process, we employed *operando* attenuated total reflectance‐Fourier transform infrared (ATR‐FTIR) spectroscopy.^[^ ^21^ ^]^ This technique provided real‐time insights into the voltage‐dependent oxidation of double bonds. ATR‐FTIR tests performed on the Mo─N─O catalyst over a voltage range of 0 to −1 V (vs the reversible hydrogen electrode, RHE) revealed a sharp peak at 1658 cm^−1^, attributed to the ─C═O stretching vibration of benzaldehyde (Figure 4c). The intensity of this peak initially increased, then decreased, and finally increased again with rising voltage. Additionally, a broad peak at 3510 cm^−1^, assigned to adsorbed ‐OH on the catalyst's active site, exhibited a trend consistent with previously reported findings.^[^ ^22^ ^]^ This behavior suggests that at low voltages, 2*e* ^−^ ORR favors the formation of ^*^OOH, accelerating oxidation and increasing adsorbed products at the catalyst‐electrolyte interface. At higher voltages, 4*e* ^−^ ORR dominates, leading to O═O bond cleavage and subsequent formation of ^*^OH through electron and proton acceptance. Consequently, the voltage modulation enables precise control over the ORR pathway, facilitating selective oxidation of double bonds in the presence of aldehydes.

To gain a deeper understanding of the underlying reaction mechanisms, we proposed an electrochemically mediated pathway involving the ^*^OOH radical, aimed at elucidating its highly selective mechanism for oxidizing C═C double bonds (Figure 4d; Figure S34, Supporting Information). The proposed reaction mechanism begins with the ^*^OOH radical abstracting a hydrogen atom from the aldehyde group of cinnamaldehyde (1), generating a highly reactive aldehyde radical (2). This radical then captures an oxygen molecule to form an organic peroxide radical (3). Subsequently, the radical intermediate (3) undergoes intramolecular cyclization, forming a tetrahydrofuran structure (4) accompanied by the intramolecular transfer of the radical site. During this process, the *p*‐*Π* conjugation effect promotes the generation of a benzyl‐type radical, which further captures oxygen to produce a new tetrahydrofuran intermediate (5). As the reaction progresses, intermediate (5) undergoes structural rearrangement to transform into intermediate (6). Due to the weak bond energy of the O─O bond and the high ring strain of the tetrahydrofuran, the O─O bond and C─C bond in intermediate (6) are readily cleaved, resulting in the formation of benzaldehyde (7). Concurrently, another radical (8) serves as an initiator, triggering a new reaction cycle that ultimately yields products including benzaldehyde (7) and glyoxal (9). The insights gained from this study provide a comprehensive understanding of the selective oxidation mechanism facilitated by Mo─N─O catalysts. The proposed pathway elucidates how ^*^OOH radicals effectively mediate the selective cleavage of C═C double bonds without affecting other functional groups, highlighting the potential for Mo─N─O in various catalytic applications. Further investigations into the scalability and applicability of Mo─N─O in other oxidation reactions could pave the way for its broader utilization in the field of fine chemicals and green chemistry.

**Figure 5:**
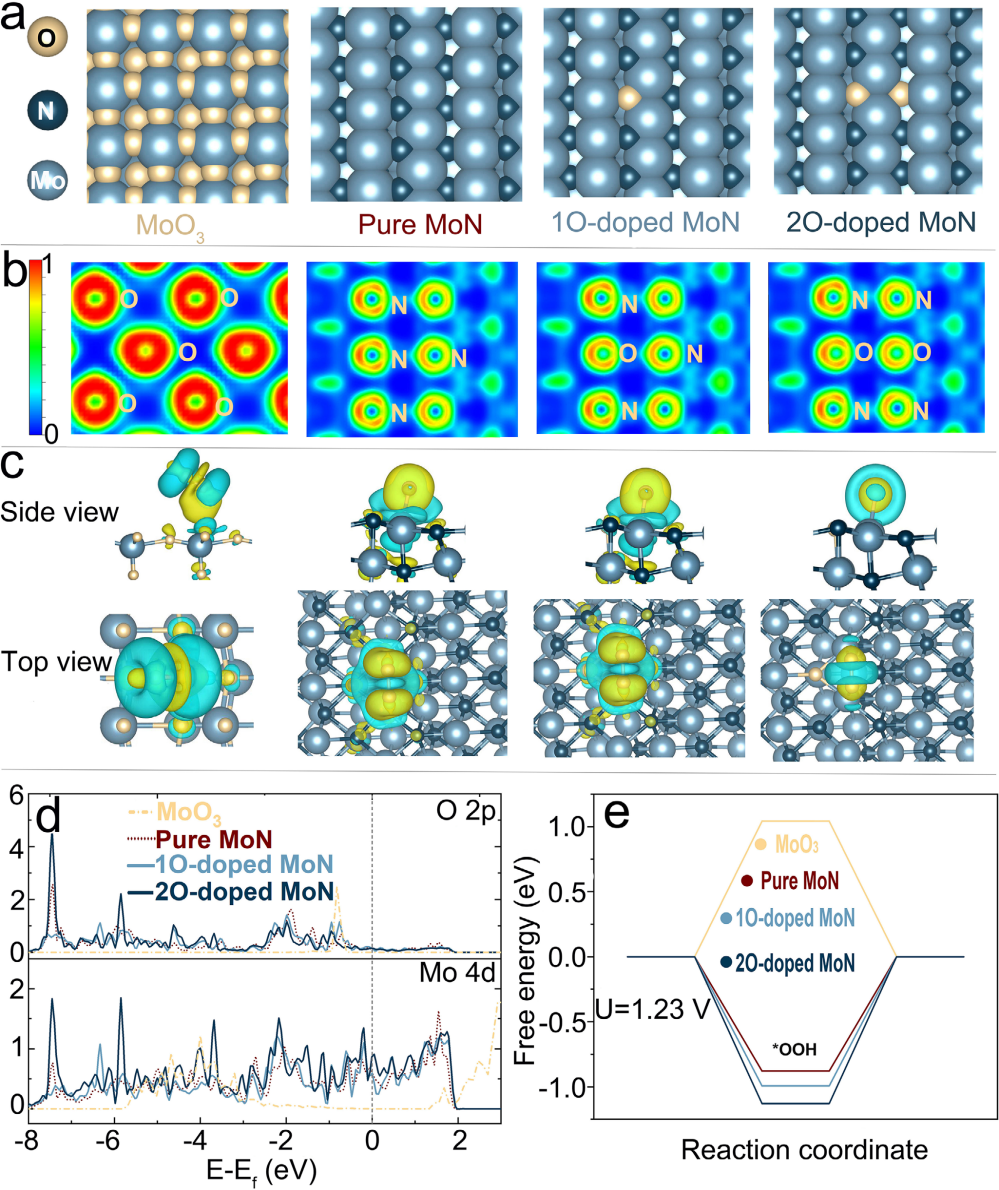
DFT calculations: a) Atomic models used for DFT simulations; b) Comparison of ELF results for MoO_3_, pure MoN, 1O‐doped MoN and 2O‐doped MoN (from left to right); c) Images of charge density difference for MoO_3_, pure MoN, 1O‐doped MoN and 2O‐doped MoN (from left to right). Yellow and cyan isosurfaces represent regions of electron gain and loss, respectively; d) Calculated DOS; e) Comparison of the free energy for Mo‐^*^OOH intermediates.

In the selective oxidation of double bonds in the presence of aldehyde groups, experimental evidence highlights the indispensable role of the ^*^OOH radical. To investigate the intrinsic mechanisms underlying Mo‐based catalysts and their catalytic activities—particularly the generation of the ^*^OOH radical—we employed density functional theory (DFT) calculations. These calculations included analyses of the electron localization function (ELF), differential charge density, density of states (DOS), and reaction energy barriers. For comparative evaluation, molecular models such as MoO_3_ (precursor), pure MoN, and two oxygen‐doped MoN variants (1O‐doped MoN and 2O‐doped MoN) were studied to elucidate structural differences (**Figure** **5**a). ELF analysis reveals highly localized electron distributions around O atoms, characterized by pronounced ELF isosurface regions, indicating strong electron localization and resistance to delocalization. Oxygen doping in MoN significantly alters the electronic structure, as seen in 1O‐doped and 2O‐doped MoN, compared to pure MoN (Figure 5b). This suggests that oxygen doping induces structural and electronic modifications in MoN, thereby enhancing its catalytic properties. Upon O_2_ adsorption onto the catalyst surface, differential charge density maps indicate electron accumulation primarily on Mo atoms, with pronounced electron transfer on non‐metal atoms (O and N) (Figure 5c). By tuning the O/N ratio, the electronic environment at Mo active centers and their interactions with reactants can be effectively modulated.

To further understand the electronic interactions between catalytic sites and reactants, we calculated the DOS of Mo 4*d* and O 2*p* orbitals for the four Mo‐based catalysts during O_2_ adsorption (Figure 5d). Compared to MoO_3_ and pure MoN, the 1O‐doped and 2O‐doped MoN models exhibit greater overlap between Mo 4*d* and O 2*p* states at binding energy levels. This overlap indicates that oxygen doping enhances electron delocalization and strengthens Mo 4*d*–O 2*p* orbital interactions, thereby improving catalytic activity. Furthermore, the DOS distribution near the Fermi level for 1O‐doped and 2O‐doped MoN suggests superior electron transfer capabilities, correlating with increased catalytic efficiency. Since catalytic activity in the 2*e* ^−^ ORR depends on Mo‐^*^OOH adsorption energy, we compared the Gibbs free energies of the four catalysts at U = 1.23 V to elucidate the observed higher activity of O‐doped MoN in 2*e* ^−^ ORR (Figure 5e). The results indicate that MoO_3_ has positive adsorption energy, making the adsorption process endothermic and unfavorable for catalysis. In contrast, MoN, 1O‐doped MoN, and 2O‐doped MoN exhibit negative adsorption energies, with the O‐doped variants showing larger absolute values. This implies stronger interactions between O‐doped MoN and ^*^OOH, facilitating catalytic reactions.

## 3 Conclusion

This study presents a groundbreaking approach to the selective oxidation of double bonds in the presence of aldehyde groups, addressing longstanding challenges in synthetic organic chemistry. Utilizing an electrochemical method with Mo─N─O catalysts, cinnamaldehyde was successfully transformed into benzaldehyde with exceptional selectivity and complete conversion under mild conditions. The method capitalizes on the generation of ^*^OOH radicals, which effectively target double bonds while preserving the aldehyde groups. This capability addresses a critical limitation of conventional oxidation techniques, which often require harsh conditions and yield a mixture of byproducts. By resolving key inefficiencies in existing strategies, this work establishes a strong foundation for advancing selective oxidation techniques. The findings not only expand the potential applications of electrochemical oxidation but also provide new opportunities for developing sustainable chemical processes.

## Conflict of Interest

The authors declare no conflict of interest.

## Author Contributions

Z.L., X.Z., and J.L. contributed equally to this work.

## Supporting information



Supporting Information

Supporting Information

## Data Availability

The data that support the findings of this study are available from the corresponding author upon reasonable request.
