# Supplementary material for: Sustainable Electrochemical Strategy for Selective Double Bond Oxidation in the Presence of Aldehyde Groups with Mo─N─O Catalysts
Source: Adv Sci (Weinh). 2025 Jun 23;12(36):e06584. doi: 10.1002/advs.202506584 (PMC12463080; doi:10.1002/advs.202506584)
Supplement: Supplementary file 1 — Supporting Information [file ADVS-12-e06584-s001.pdf]

ADVANCED SCIENCE Open Access

Supporting Information

for *Adv. Sci.*, DOI 10.1002/advs.202506584

Sustainable Electrochemical Strategy for Selective Double Bond Oxidation in the Presence of Aldehyde Groups with Mo—N—O Catalysts

Zhirong Li, Xun Zhang, Jiao Liu, Zhaomin Hao, Tengfei Zhang, Yang Yang, Yitong Wang, Santhosh Kamaraj, Jianrong Zeng, Qingsong Dong*, Cuiping Zhai*, Wuping Liao* and Shuyan Song**

Supporting Information

Sustainable Electrochemical Strategy for Selective Double Bond Oxidation in the Presence of Aldehyde Groups with Mo-N-O Catalyst

Zhirong Li,[a, b] *Xun Zhang,*[b] *Jiao Liu,*[a, b] *Zhaomin Hao,*[b, *] *Tengfei Zhang,*[a, b] *Yang Yang,*[a, b] *Yitong Wang,*[a, b] *Santhosh Kamaraj,*[a, b] *Jianrong Zeng,*[c] *Qingsong Dong,*[a, *] *Cuiping Zhai,*[a, *] *Wuping Liao*[b, *] *Shuyan Song*[d, *]

[a]College of Chemistry and Molecular Sciences, Henan University, Kaifeng, P. R. China
[b]Ganjiang Innovation Academy, Chinese Academy of Sciences, Ganzhou, P. R. China
[c]Shanghai Synchrotron Radiation Facility, Shanghai Advanced Research Institute, Chinese Academy of Sciences, Shanghai, P. R. China
[d]Changchun Institute of Applied Chemistry, Chinese Academy of Sciences, Changchun 130022, P. R. China

Corresponding Author:
E-mail: zmhao@gia.cas.cn, songsy@ciac.ac.cn

Content

Methods 4

Synthesis of Mo-N-O 4

Characterization 4

Electrochemical measurements 6

Figure S1. Comparison between traditional approaches and the present work for achieving selective oxidation of double bonds in the presence of aldehyde groups 9

Figure S2. List of potential oxidation products of cinnamaldehyde 10

Figure S3. Schematic elucidation of the electrochemical reaction in this work 11

Figure S4. SEM image of the Mo-O 12

Figure S5. SEM image of the Mo-N 13

Figure S6. SEM image of the Mo-N-O 14

Figure S7. XRD pattern of Mo-N-O, Mo-N and Mo-O 15

Figure S8. EDS elemental mapping images of Mo-O 16

Figure S9. EDS elemental mapping images of Mo-N 17

Figure S10. EDS elemental mapping images of Mo-N-O 18

Figure S11. Comparison of EPR spectra for Mo-N-O, Mo-N and Mo-O 19

Figure S12. The average oxidation state of Mo^{x+} in Mo-N-O and Mo-N as determined by *K*-edge XANES 20

Figure S13. The fitting curve of *K*-space for Mo-foil 21

Figure S14. The fitting curve of *K*-space for Mo-O 22

Figure S15 The fitting curve of *K*-space for Mo-N 23

Figure S16. The fitting curve of *K*-space for Mo-N-O 24

Figure S17. Wavelet transforms (WT) contour plots of the Mo-foil 25

Figure S18. WT contour plots of the Mo-O 26

Figure S19. WT contour plots of the Mo-N 27

Figure S20. WT contour plots of the Mo-N-O 28

Figure S21. Linear sweep voltammetry of rotating ring-disk electrode (RRDE) measurement for Mo-N-O at 900 rpm in O_2-saturated electrolyte at 5 mV s^{-1} 29

Figure S22. Linear sweep voltammetry of RRDE measurement for Mo-N at 900 rpm in O_2-saturated electrolyte at 5 mV s^{-1} 30

Figure S23. The corresponding average number of transferred electrons (*n*) 31

Figure S24. The Selectivity of $2e^-$ ORR for Mo-N and Mo-N-O 32

Figure S25. The concentrations and faradaic efficiencies of $2e^-$ ORR at different operating currents for 1 h by a two-PEM electrolytic cell 33

Figure S26 Comparison of concentration and selectivity in this work and previously reported works. 34

Figure S27 Comparison of Faraday efficiency and concentration of Mo-O, Mo-N and Mo-N-O catalysts at a current of -200 mA 35

Figure S28 Comparison of XRD patterns before and after electrolysis using Mo-N-O 36

Figure S29 Comparison of the ^{1}HNMR spectrum of 4-pentenal before and after the reaction37

Figure S30 Comparison of the ^{1}HNMR spectrum of α-methylstyrene ketone before and after the reaction 38

Figure S31 Comparison of the ^{1}HNMR spectrum of cinnamic acid before and after the reaction. 39
Figure S32 Comparison of the ^{1}HNMR spectrum of Citral before and after the reaction 40
Figure S33 Comparison of the ^{1}HNMR spectrum of 2-butenal before and after the reaction41
Figure S34 Comparison of carbon dioxide content between raw gas and post-reaction as measured by GC 42
Table S1 Comparison of the selectivity and yields of various organic substrates 43

Methods

Synthesis of Mo-N-O

The synthesis of Mo-N-O involves a two-step process, starting with the preparation of the Mo-N precursor. First, 0.5 g of commercial molybdenum trioxide (MoO_3) is precisely weighed using an analytical balance and evenly distributed in a clean porcelain boat. The boat is partially covered to permit ammonia gas entry and placed at the center of a tube furnace.

To initiate the reaction, the furnace is purged with ammonia gas and gradually heated to 700°C at a controlled rate of 5°C/min, with a continuous NH_3 flow of 40 sccm. This temperature is held for 2 hours to ensure complete ammoniation of MoO_3. Upon completion, the sample is allowed to cool to room temperature under an ammonia atmosphere, resulting in black Mo-N powder.

In the subsequent oxidation step, the Mo-N powder is transferred to a crucible and heated in preheated air at 300°C for 5 minutes using a muffle furnace. This process yields the final Mo-N-O sample.

Characterization

The crystalline structure of the sample was characterized using a Bruker D8 Focus X-ray diffractometer (XRD) equipped with Cu Kα radiation (λ = 0.154 nm), operated at 40 kV and 40 mA. X-ray photoelectron spectra (XPS) were obtained at room temperature using a VG ESCALAB MK (VK Company, UK) with an Al Kα X-ray source operating at 12 kV and 20 mA. Energy dispersive spectroscopy (EDS) data were collected using a field emission scanning electron microscope (SEM) equipped with an energy-dispersive X-ray spectroscopy detector to analyze the elemental composition. Electron paramagnetic resonance (EPR) measurements for both the catalyst and the captured *OOH radical were performed using a Bruker EMXplus6/1. Raman spectra were obtained using a confocal laser Raman spectrometer (Edinburgh,

RM5) with a 785 nm laser. Transmission electron microscopy (TEM) was conducted on an FEI Tecnai G2 S-Twin instrument, featuring a field emission gun operating at 200 kV. Nuclear magnetic resonance (NMR) spectra were recorded on a Bruker AVANCE ONE 400 MHz spectrometer to examine the selective oxidation products of α, β-unsaturated aldehydes in $CDCl_3$, using tetramethylsilane (TMS) as an internal standard. The selectivity of organic products was calculated based on HNMR results. Peak integrals (*I*) scale linearly with the molar amount (*X*) and proton count (*N*), as defined in equations (1)–(4):

$$\frac{I_i}{N_i} \propto X_i \text{ ....}(1)$$

$$\frac{I_p}{N_p} \propto X_p \text{ ....}(2)$$

$$\mathrm{X_i} = \sum_x I_i / N_i = I_1 / N_1 + I_2 / N_2 + I_3 / N_3 + \cdots\cdots + I_x / N_x \text{ ....}(3)$$

$$Selectivity(\%) = \frac{X_p}{X_i + X_p} \bullet 100\% = \frac{I_p / N_p}{\sum_x (I_i / N_i) + I_p / N_p} \bullet 100\% \text{ ....}(4)$$

Here, I_p, X_p and N_p correspond to the benzaldehyde signal, while I_i, X_i, and N_i refer to each byproduct. Prior to integration, we confirmed all relevant chemical shifts and selected non-overlapping resonances. X-ray absorption near-edge structure (XANES) and extended X-ray absorption fine structure (EXAFS) analyses were performed at the Shanghai Synchrotron Radiation Facility. Gas chromatography (GC) was conducted using a Shimadzu Rtx-5MS column with helium as the carrier gas and an inlet temperature of 280°C. ATR-FTIR spectra were recorded using a Thermo Scientific Nicolet iS50 FTIR spectrometer equipped with a SOFTEC in situ electrochemical characterization system (YuanGuang, Beijing, China). The measurements were performed in an H-type electrochemical cell, using a gold film deposited on a wedge-shaped silicon crystal as the working electrode. A saturated calomel electrode served as the reference electrode, and platinum was used as the counter electrode. The ink of the catalyst was prepared following the same protocol of ORR measurements. A 35 μL aliquot of the ink was drop-cast onto the center of the gold film. The anolyte consisted of 5 mL of 0.5 M H_2SO_4, while the catholyte was an

O_2-saturated solution comprising 9 mL of 0.1 M Na_2SO_4, 1 mL of DMF, and 100 μL of cinnamaldehyde. Electrochemical measurements were carried out over a potential range of 0 to –1 V *vs*. RHE. Infrared spectra were recorded in the range of 1000–4000 cm^{-1}.

Electrochemical measurements

The performance of the two-electron oxygen reduction reaction ($2e^-$ ORR) was evaluated using a rotating ring-disk electrode (RRDE) operating at 900 rpm in an oxygen-saturated 0.1 M KOH electrolyte. A platinum sheet electrode served as the counter electrode, and a saturated calomel electrode (SCE) was used as the reference electrode. The RRDE (AFE7R9GCPT, Pine Research Instrumentation, USA) comprises a Pt ring (ring area: 0.1866 cm^2) and a glassy carbon rotation disk electrode (disk area: 0.2475 cm^2).

To assess the $2e^-$ ORR performance, 1 mg of Mo-based electrocatalysts was dispersed in a mixture of 225 μL isopropanol, 50 μL Nafion solution (5.0 wt%), and 75 μL deionized water. After sonication for 1 hour, 5.0 μL of the resulting suspension was pipetted onto the disk electrode. Before testing, all electrocatalysts were stabilized through cyclic voltammetry (CV) at a scan rate of 50 mV s^{-1}. Linear sweep voltammetry (LSV) was then conducted at a scan rate of 5.0 mV s^{-1}. The ring electrode was maintained at a constant potential of 1.20 V vs. RHE, following the procedure outlined by Jens K. Nørskov and Yi Cui (*Nat. Catal.*, **2018**, *1*, 156), to avoid ORR currents at the ring and allowing only H_2O_2 oxidation.

Next, the electrochemical experiments were conducted in a standard two-electrode system using constant current electrolysis. The opposing electrode was made of titanium mesh uniformly coated with IrO_2, while the working electrode consisted of carbon paper evenly coated with the catalyst on the back of the gas diffusion layer (GDL). For the preparation of the catalyst suspension, 5 mg of Mo-N-O electrocatalyst was dispersed in a mixture of 225 μL isopropyl alcohol, 50 μL Nafion solution (5.0 wt%), and 75 μL deionized water. After mixing the catalyst

uniformly with ultrasonic treatment for 1 hour, the resulting suspensions were sequentially sprayed onto the carbon paper.

The electrochemical testing in the two-PEM cell followed a similar approach to that described by Wang and co-workers (*Nat. Commun.* **2022**, *13*, 2880), with each cell chamber having a vertical cross-sectional area of 5 cm × 5 cm. The cathode chamber was supplied with oxygen at a flow rate of 90 sccm and a liquid flow rate of 5.4 mL/min. Both gas and electrolyte flow rates were precisely controlled using mass flowmeters and injection pumps, with the flow rate of the outlet product calibrated using a measuring cylinder. The intermediate cavity used a styryl-divinylbenzene sulfonated copolymer, Dowex 50WX8 hydrogen form (Sigma-Aldrich, 200-400 mesh), as a solid electrolyte.

The ORR electrolysis experiments were conducted at different constant currents (-10 mA, -50 mA, -100 mA, -200 mA, and -300 mA) for 1 hour under ambient pressure. During these experiments, 50 mL of pure water and oxygen were introduced into the working electrode for 1 hour. To check the long-term stability of the catalyst, the volumes of pure water, Na_2SO_4, and H_2SO_4 were increased to 500 mL. At various times, 10 mL of cathode electrolyte was extracted to measure the Faradaic efficiency.

The selectivity (*%*) and the electron transfer number (*n*) of 2e^- ORR were calculated using the following equations:

$$*\mathrm{OOH}\ (\%) = 200 \times \frac{I_r/N}{I_d + I_r/N}$$

$$n = 4 \times \frac{I_d}{I_d + I_r/N}$$

where I_r is the ring current, I_d is the disk current, and N is the current collection efficiency of the Pt ring electrode (The collection efficiency of the RRDE electrode used was determined by using the reversible $[Fe(CN)_6]^{4-}/[Fe(CN)_6]^{3-}$ system). The *OOH proof reaction was conducted as follows:

$MnO_4^- + {*}OOH + H^+ \rightarrow Mn^{2+} + O_2\uparrow + H_2O$

The selective-oxidation performance of α,β-unsaturated aldehydes was conducted by replacing pure water with a mixture of DMF and water, in which 200

μL of cinnamaldehyde was dissolved (deionized water: DMF = 2:1). The product was analyzed using GC and NMR.

Figure S1. Comparison between traditional approaches and the present work for achieving selective oxidation of double bonds in the presence of aldehyde groups

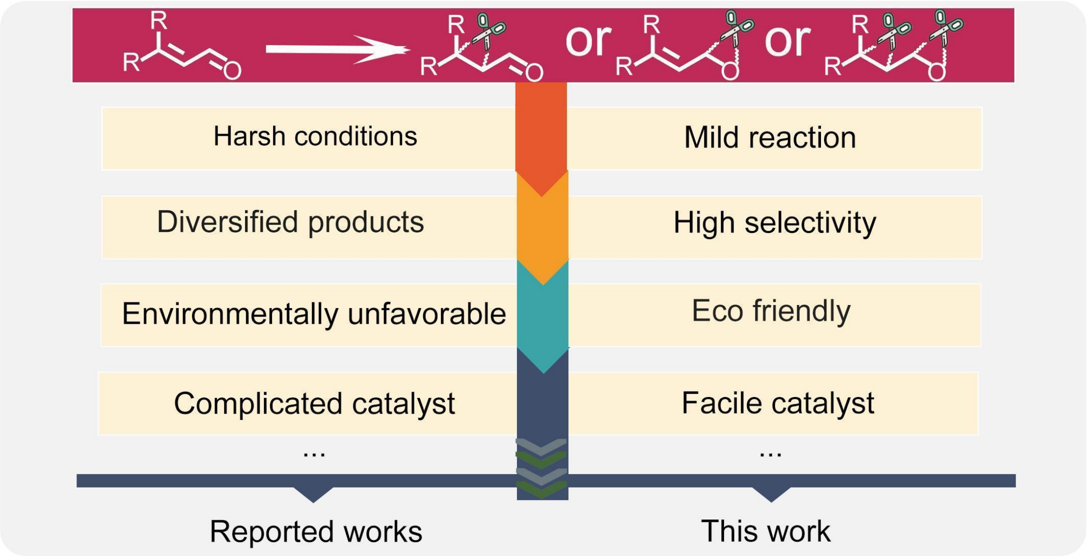

Figure S2. List of potential oxidation products of cinnamaldehyde

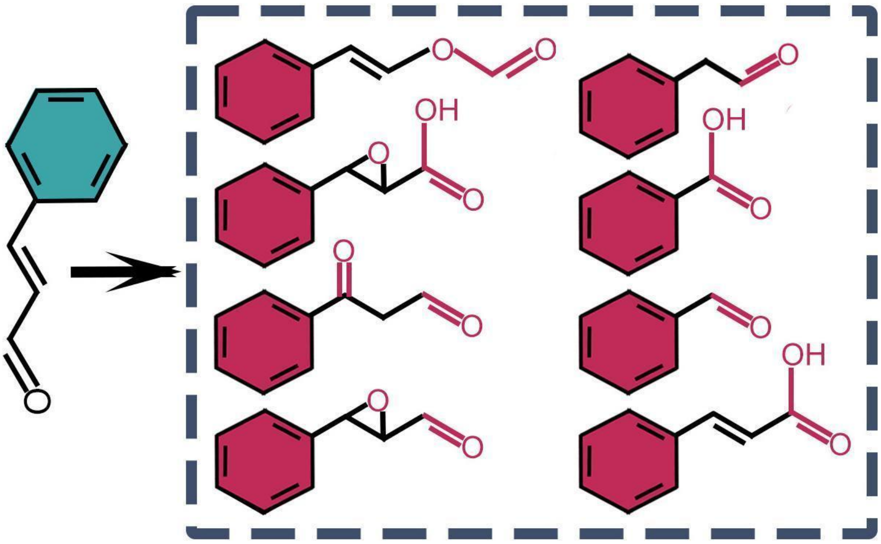

Figure S3. Schematic elucidation of the electrochemical reaction in this work

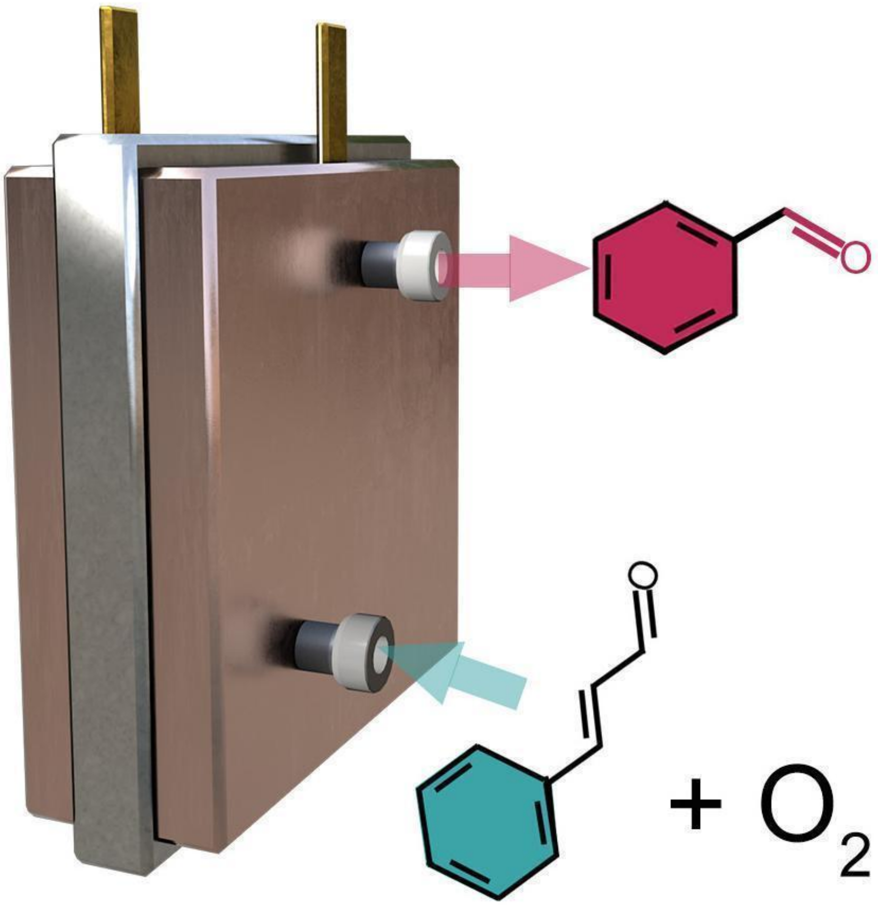

Figure S4. SEM image of the Mo-O

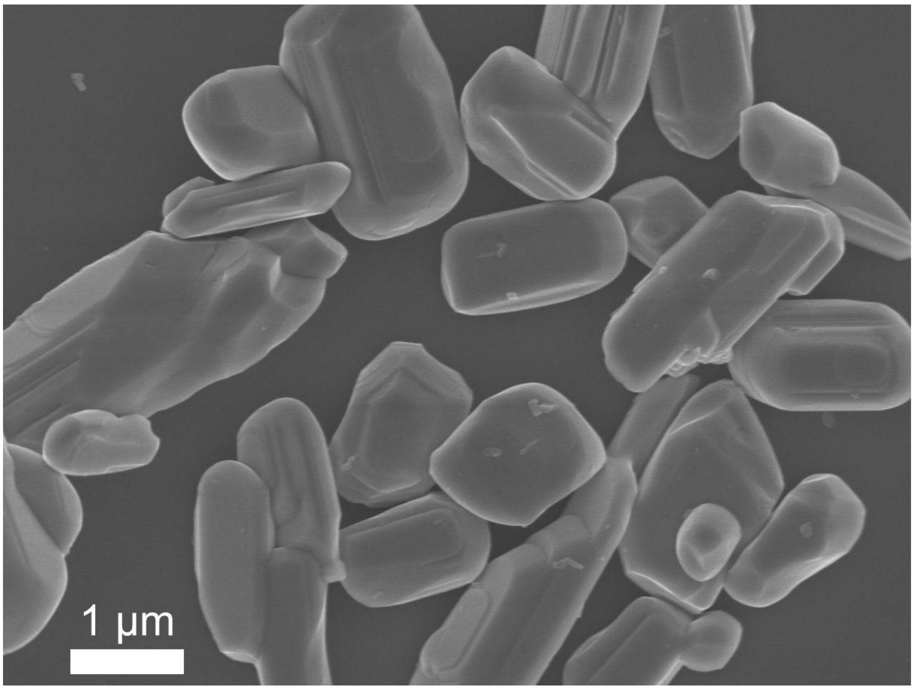

Figure S5. SEM image of the Mo-N

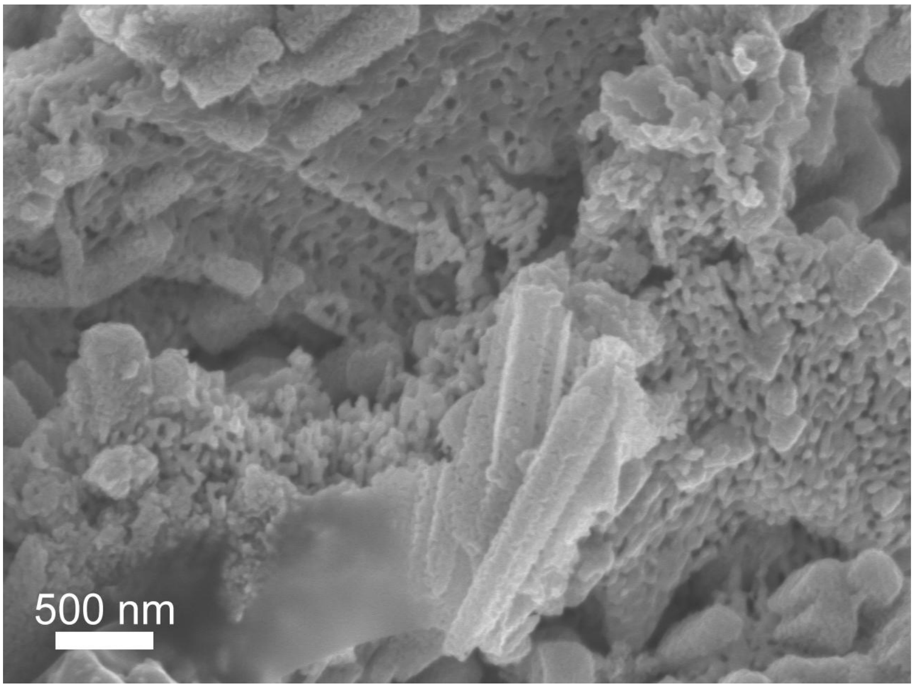

Figure S6. SEM image of the Mo-N-O

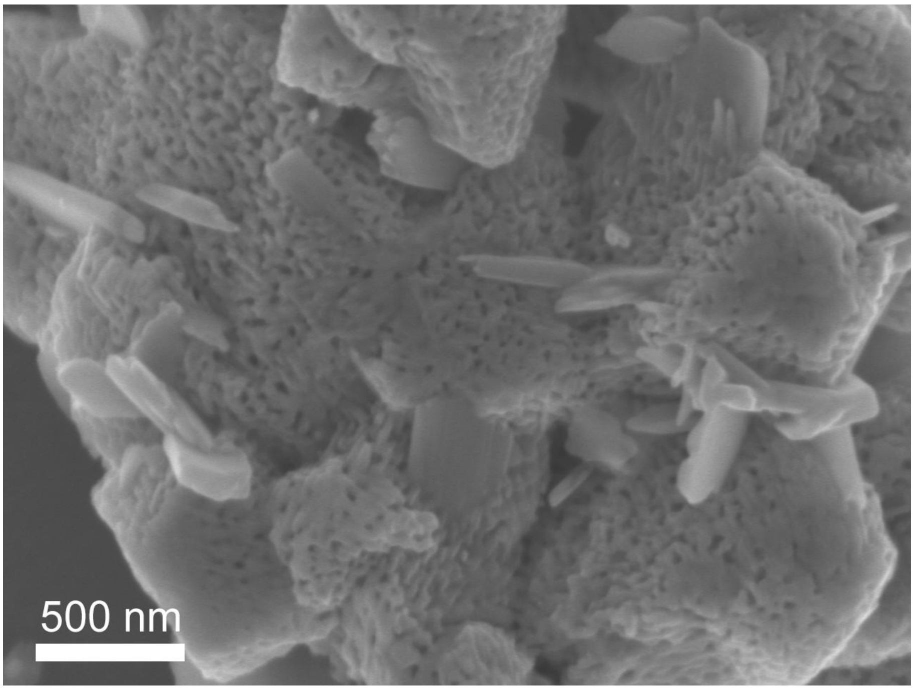

Figure S7. XRD pattern of Mo-N-O, Mo-N and Mo-O

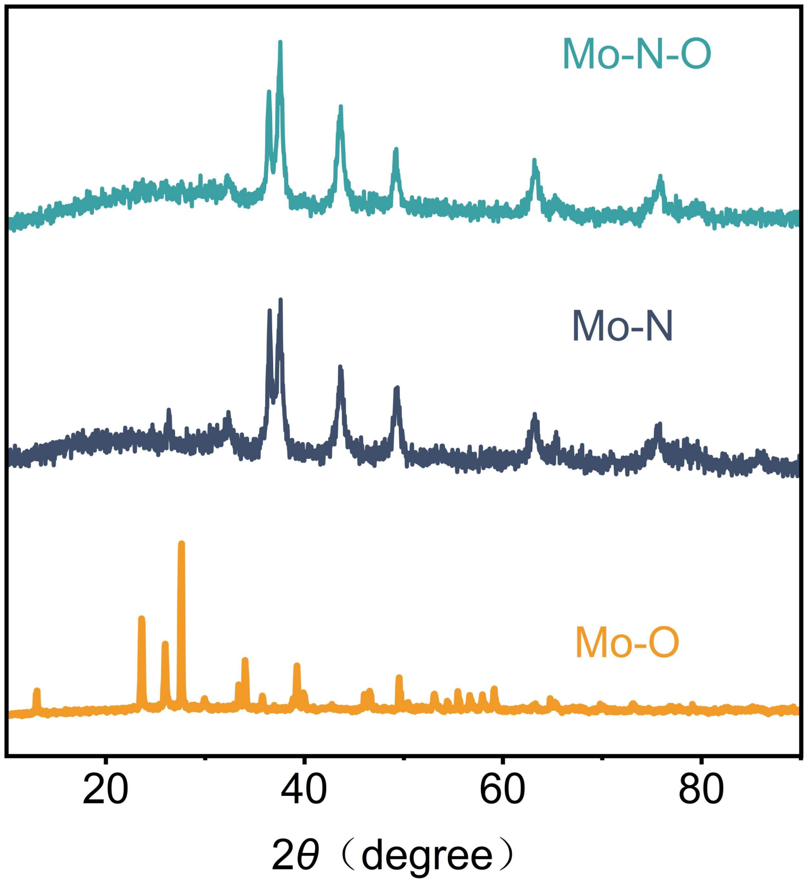

Figure S8. EDS elemental mapping images of Mo-O

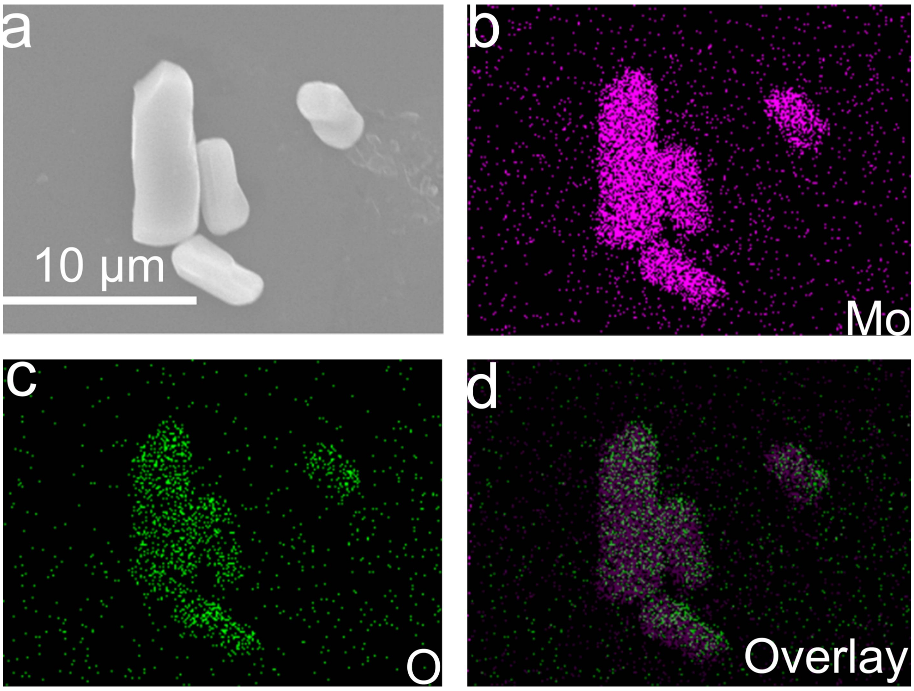

Figure S9. EDS elemental mapping images of Mo-N

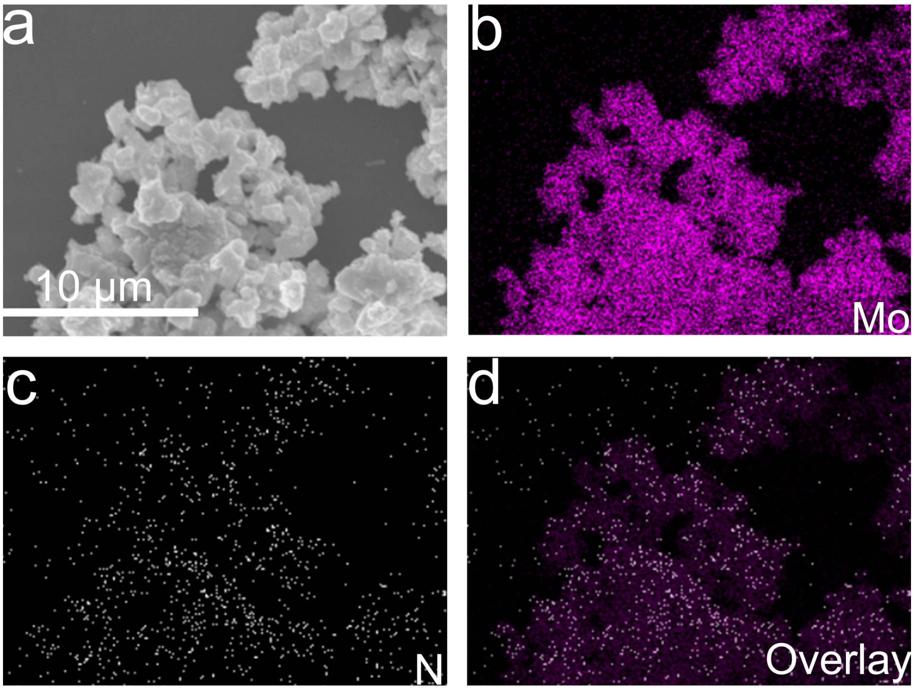

Figure S10. EDS elemental mapping images of Mo-N-O

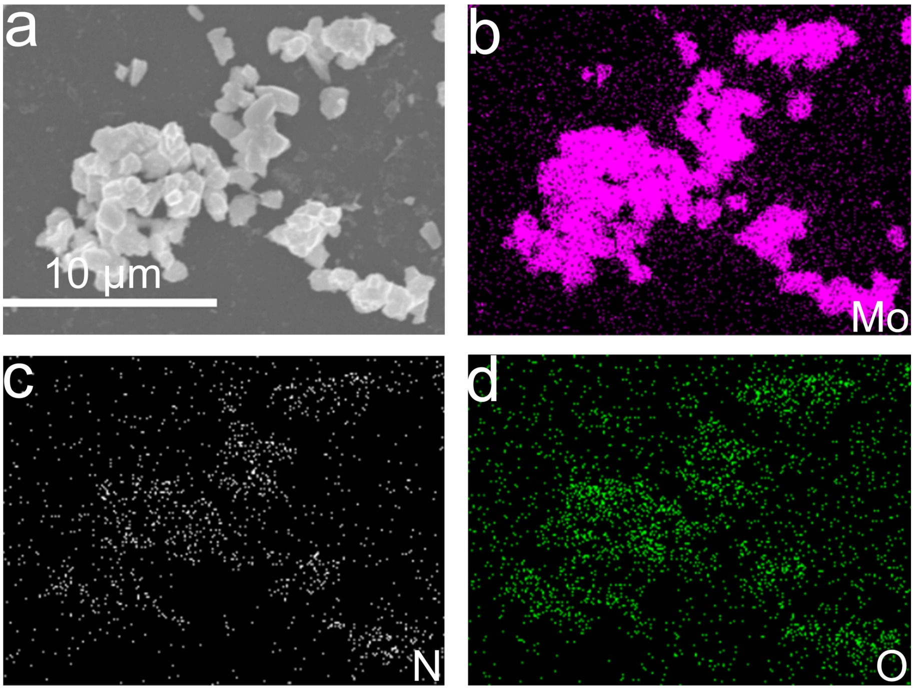

Figure S11. Comparison of EPR spectra for Mo-N-O, Mo-N and Mo-O

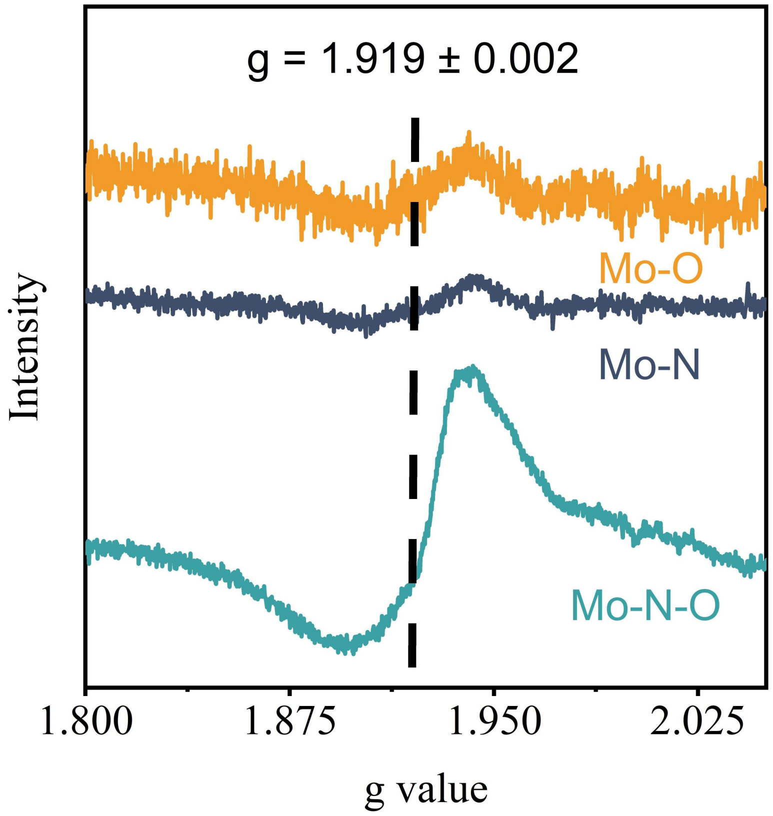

Figure S12. The average oxidation state of Mo^{x+} in Mo-N-O and Mo-N as determined by *K*-edge XANES

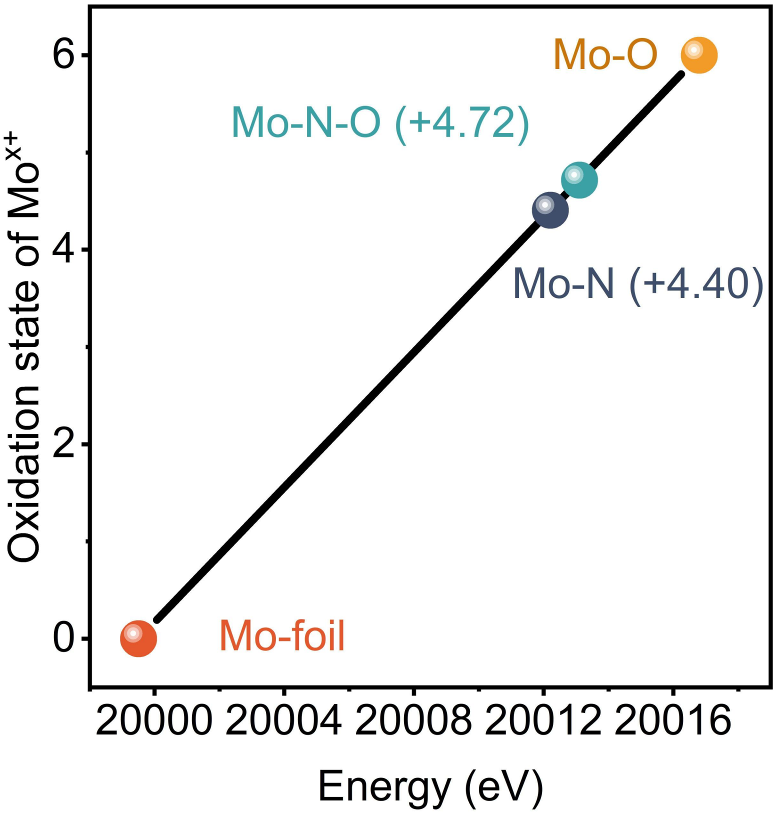

Figure S13. The fitting curve of *K*-space for Mo-foil

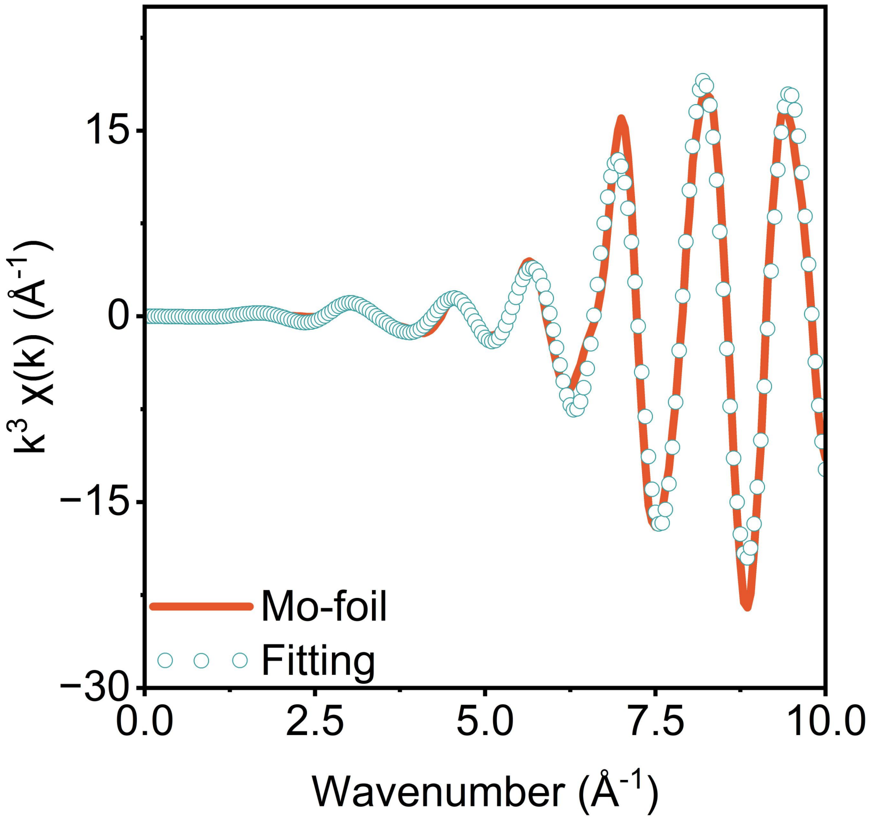

Figure S14. The fitting curve of *K*-space for Mo-O

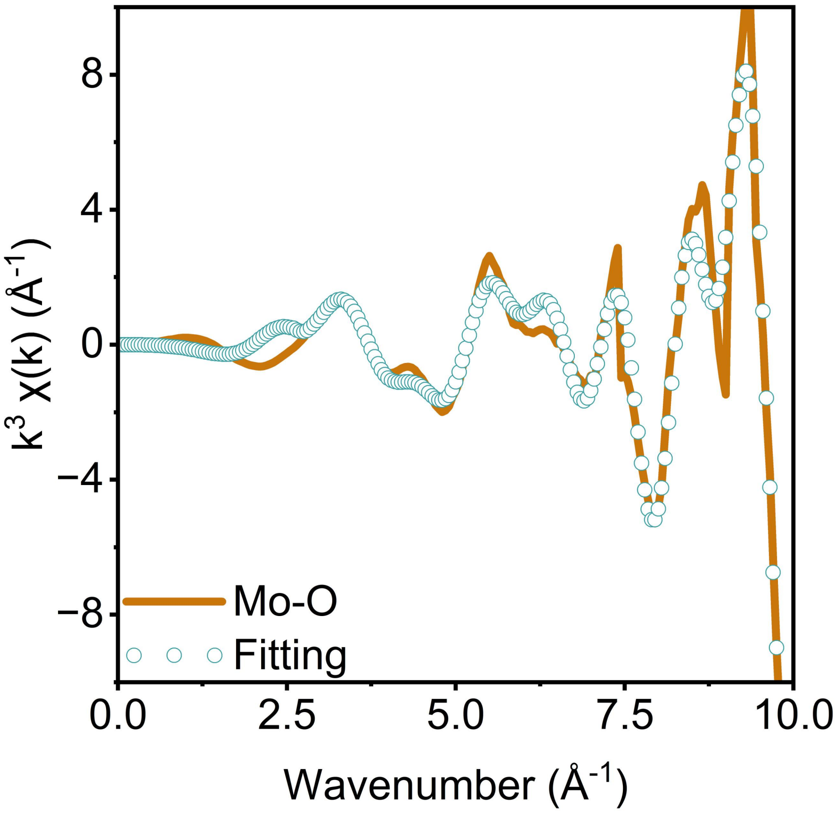

Figure S15 The fitting curve of *K*-space for Mo-N

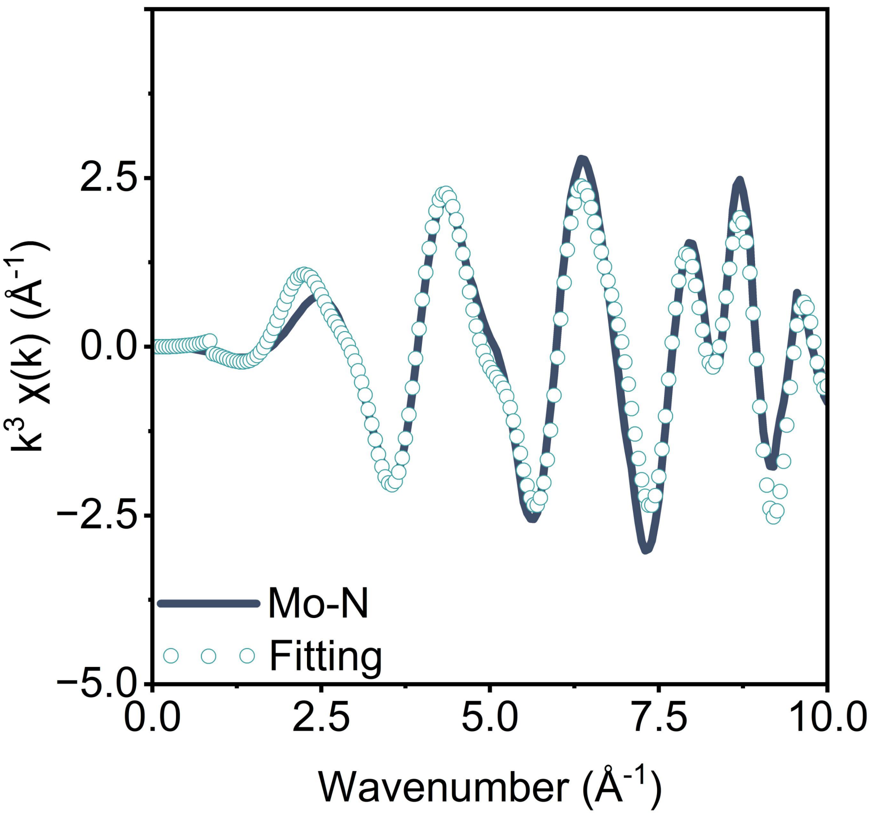

Figure S16. The fitting curve of *K*-space for Mo-N-O

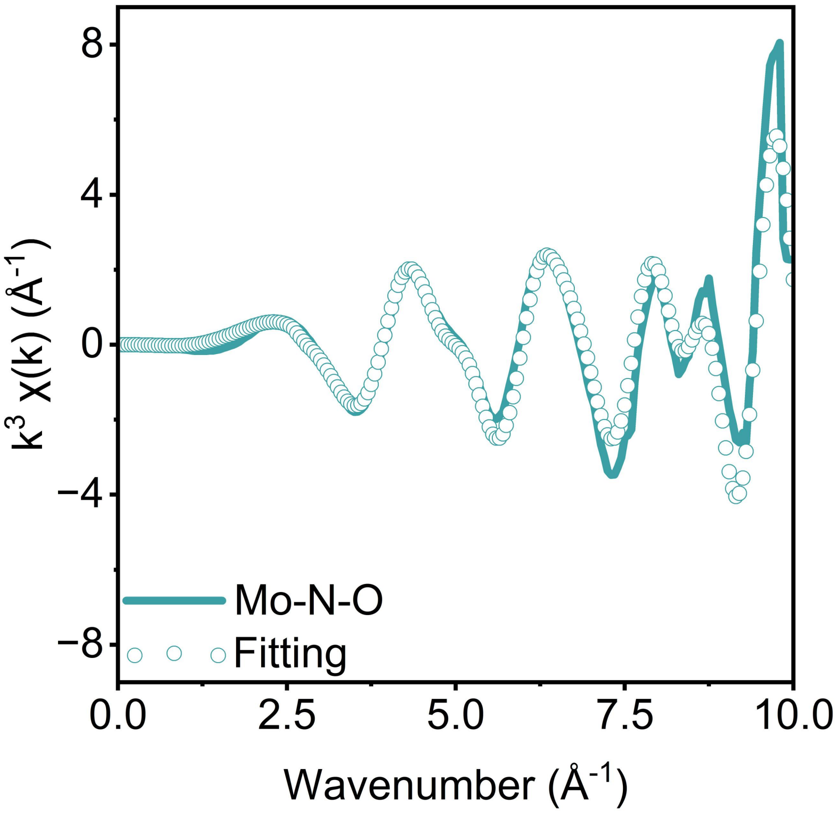

Figure S17. Wavelet transforms (WT) contour plots of the Mo-foil

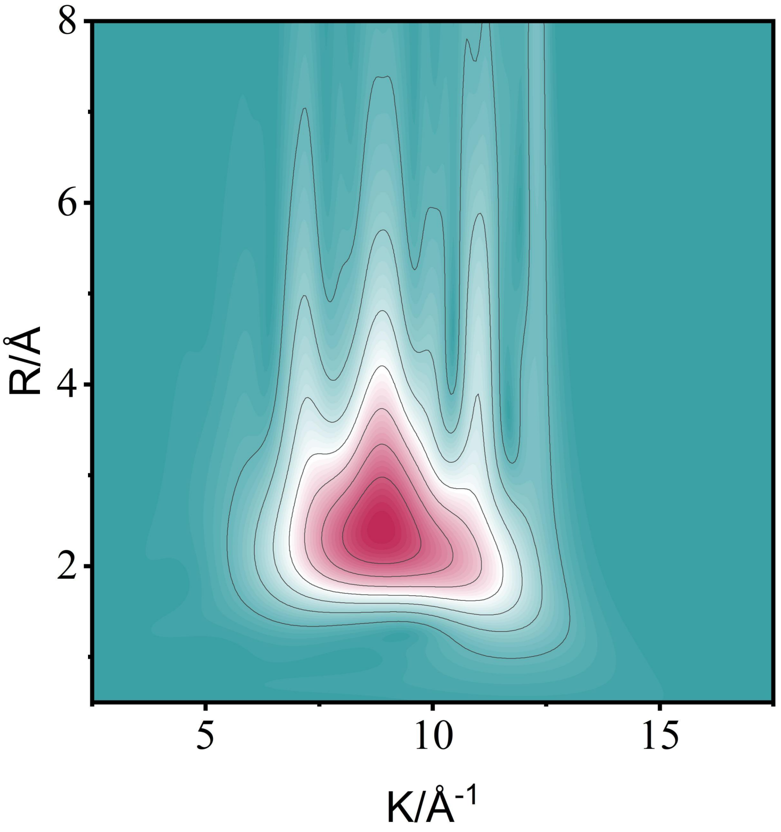

Figure S18. WT contour plots of the Mo-O

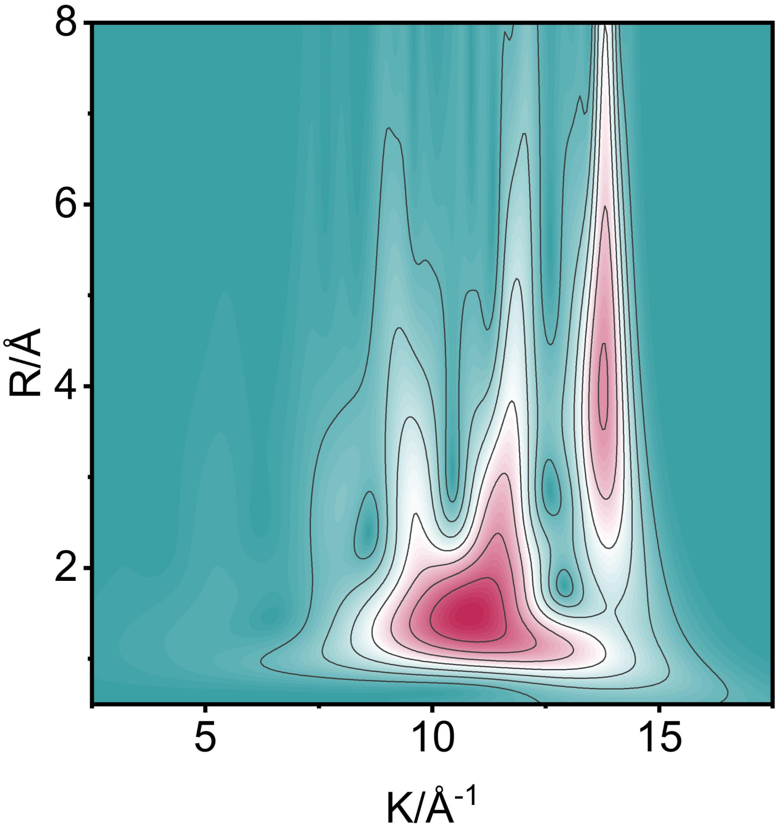

Figure S19. WT contour plots of the Mo-N

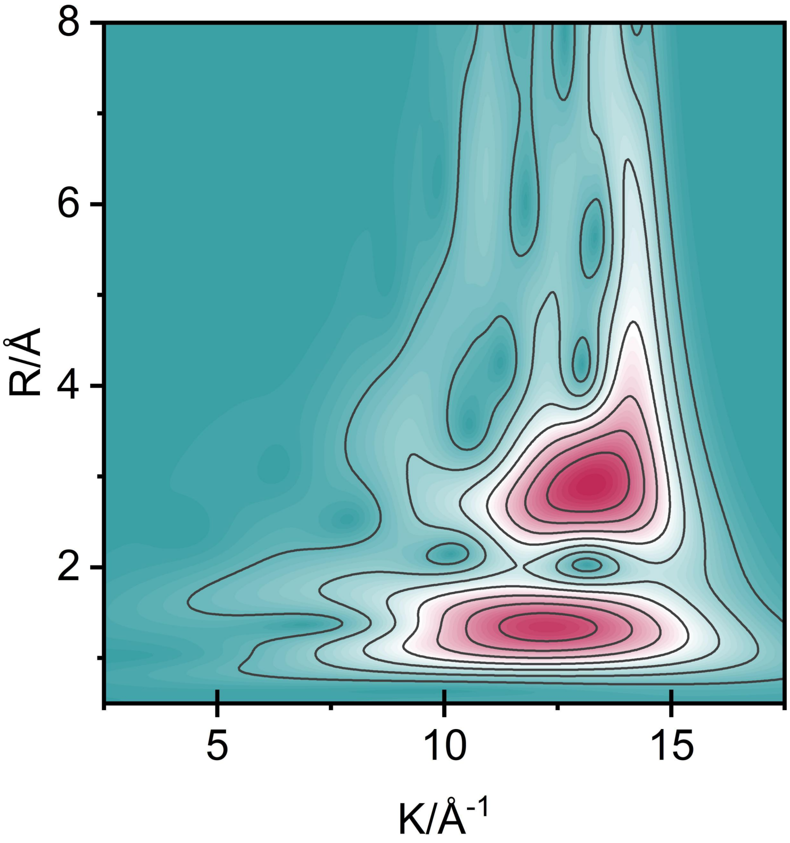

Figure S20. WT contour plots of the Mo-N-O

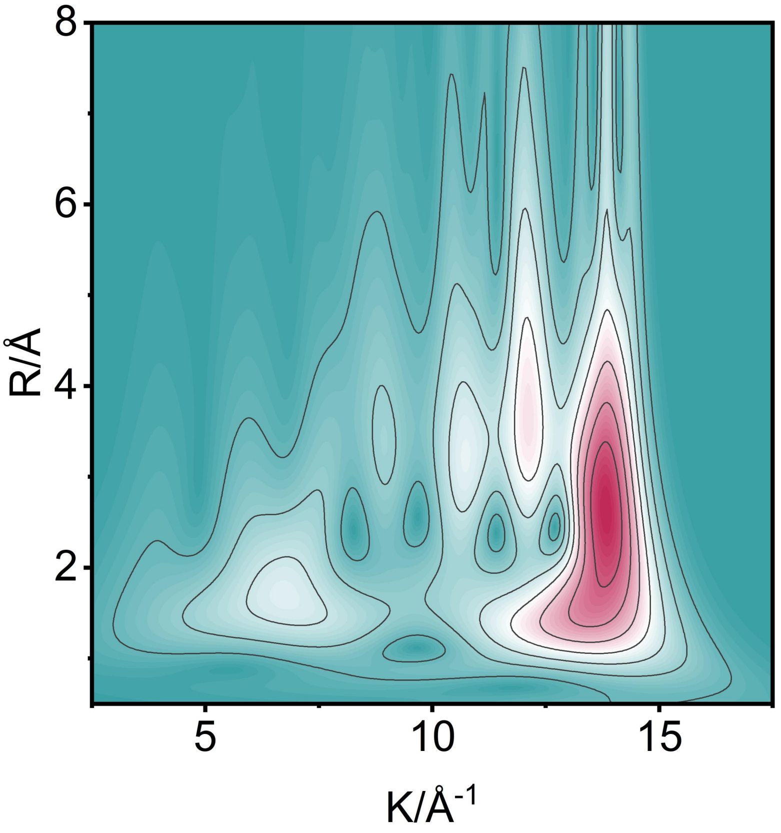

Figure S21. Linear sweep voltammetry of rotating ring-disk electrode (RRDE) measurement for Mo-N-O at 900 rpm in O_2-saturated electrolyte at 5 mV s^{-1}

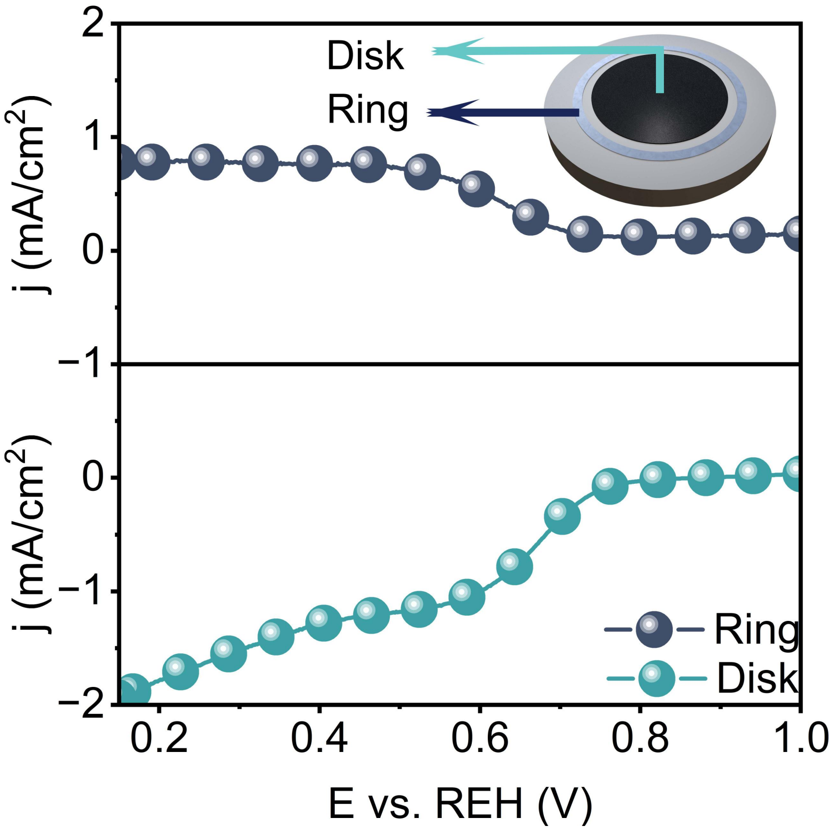

Figure S22. Linear sweep voltammetry of RRDE measurement for Mo-N at 900 rpm in O_2-saturated electrolyte at 5 mV s^{-1}

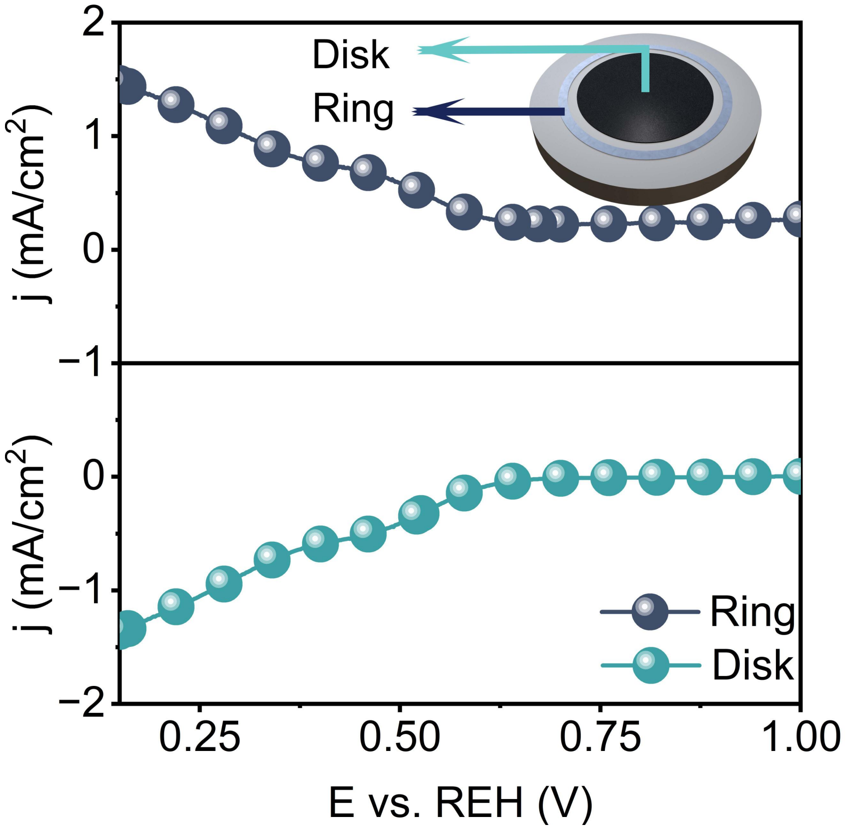

Figure S23. The corresponding average number of transferred electrons (*n*)

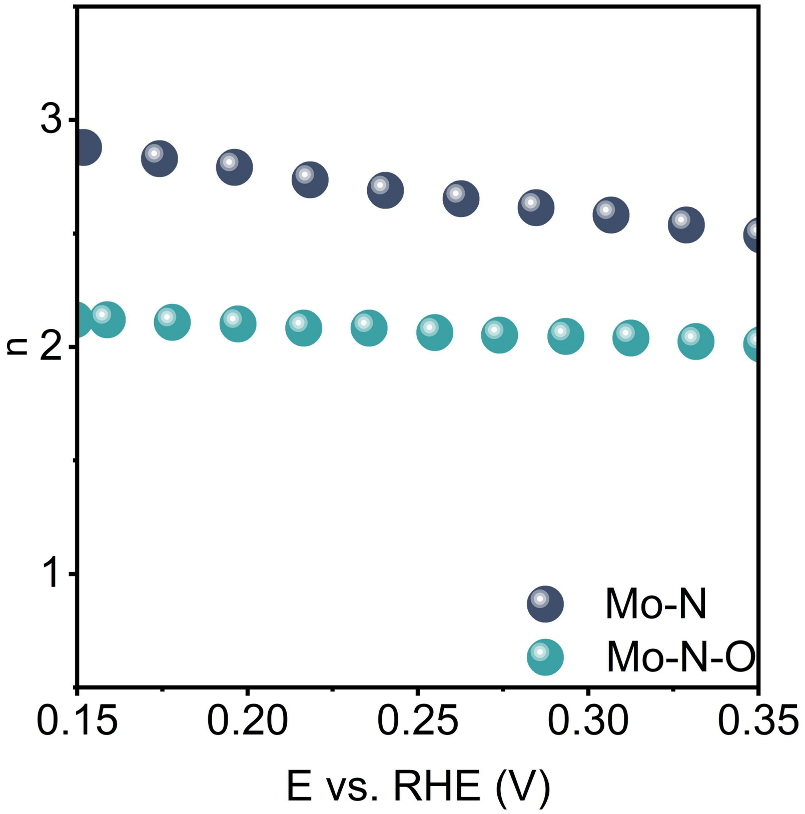

Figure S24. The Selectivity of 2e^- ORR for Mo-N and Mo-N-O

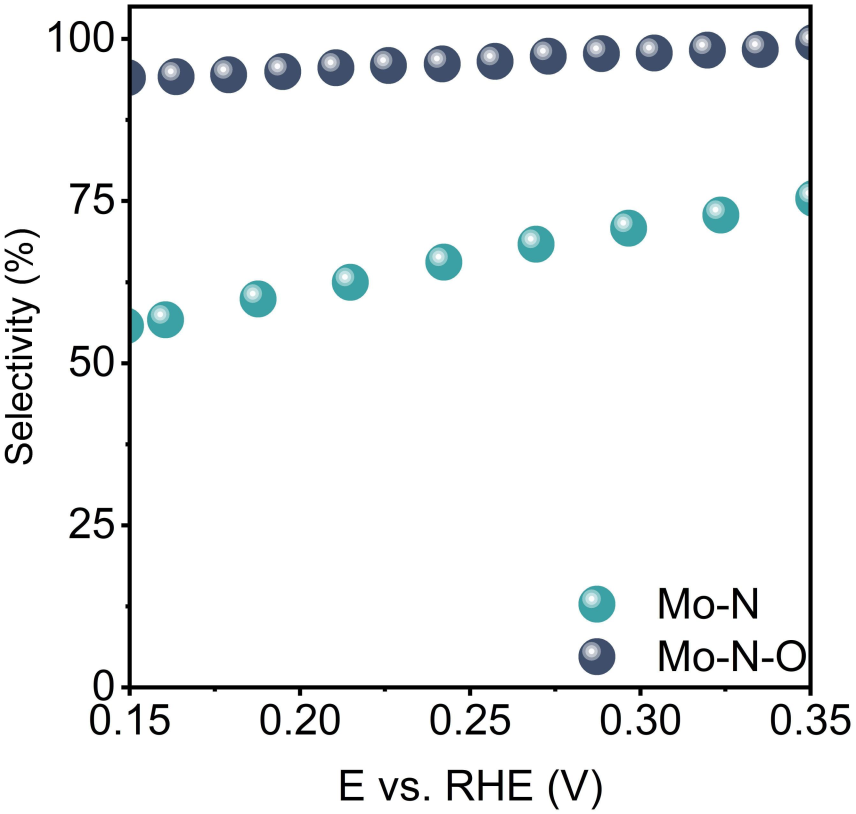

Figure S25. The concentrations and faradaic efficiencies of 2e^- ORR at different operating currents for 1 h by a two-PEM electrolytic cell

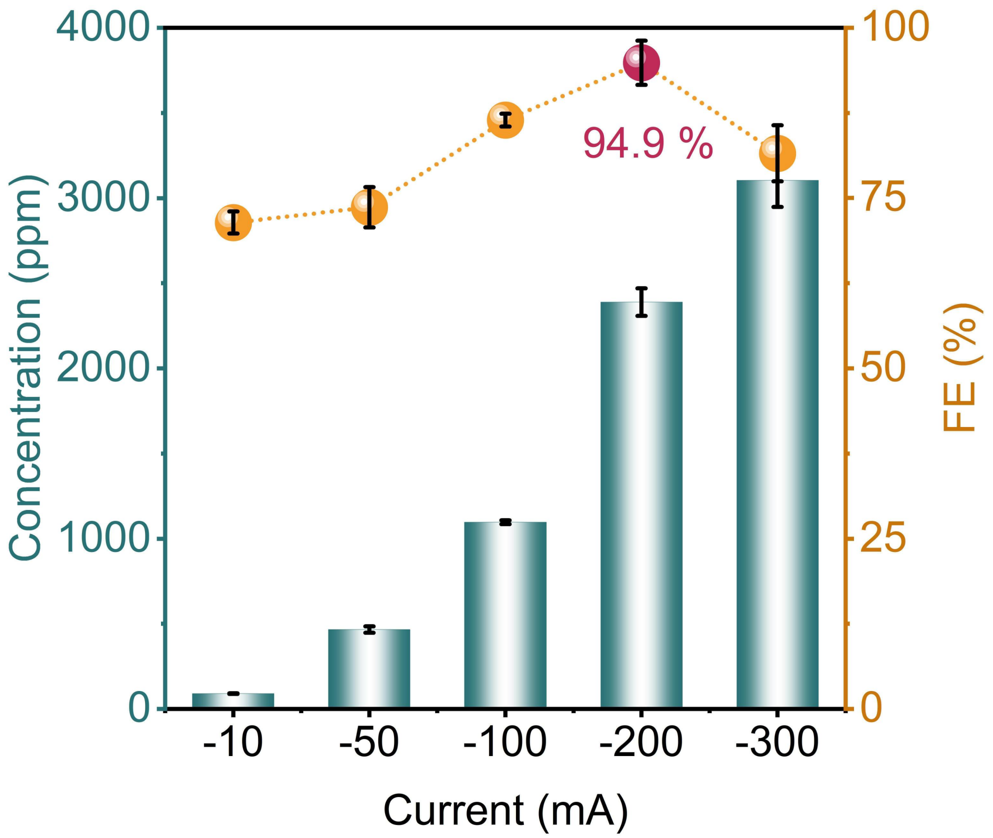

Figure S26 Comparison of concentration and selectivity in this work and previously reported works. Star, represents this work; sphere, represents N, O-codoped catalysts; triangle, represents Mo-based catalysts.

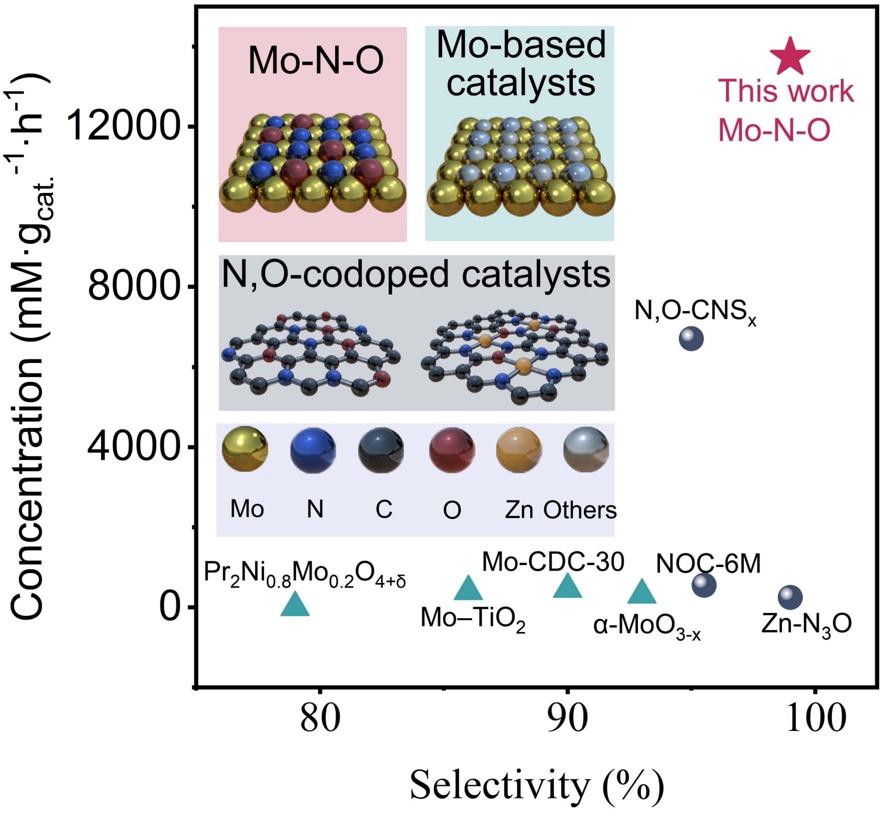

Figure S27 Comparison of Faraday efficiency and product concentration of Mo-O, Mo-N and Mo-N-O catalysts at a current of -200 mA.

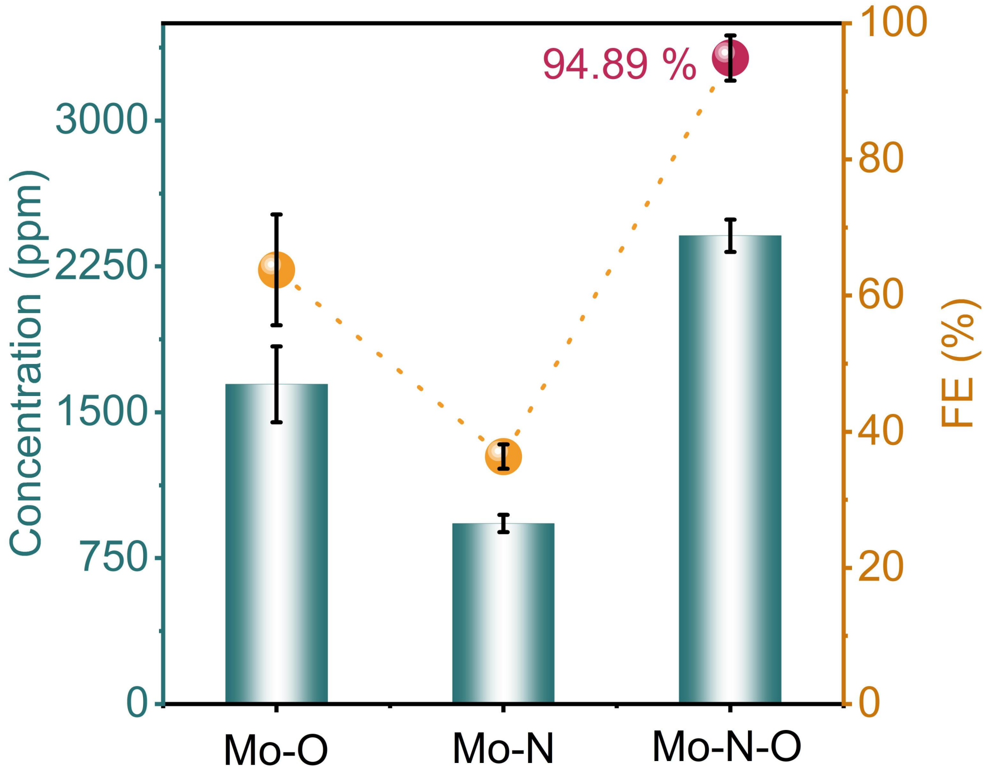

Figure S28 Comparison of XRD patterns before and after electrolysis using the Mo-N-O.

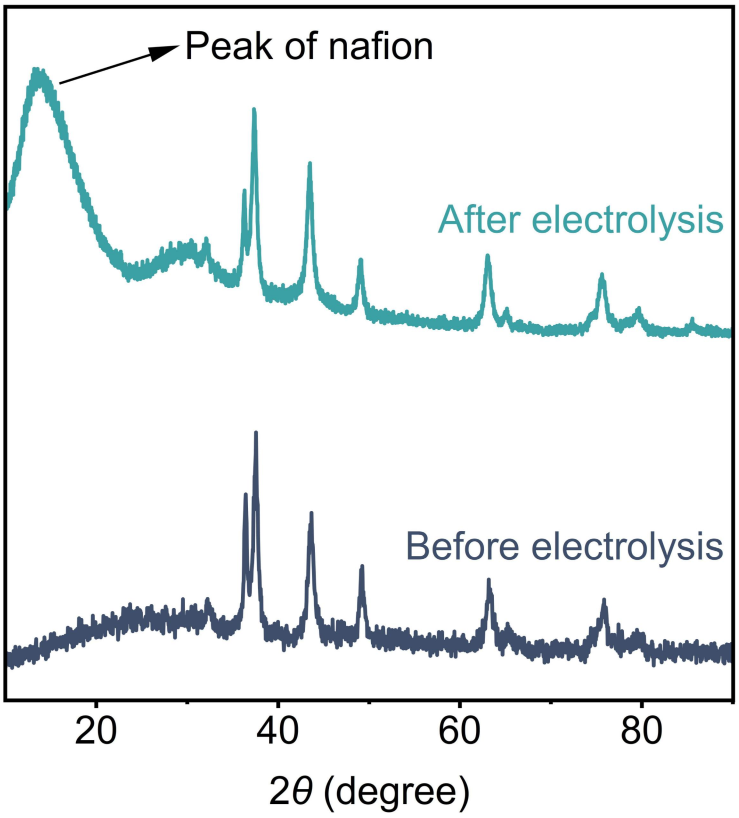

Figure S29 Comparison of the ^{1}HNMR spectrum of 4-pentenal before and after the reaction. 4-Pentenal gave succinaldehyde and sodium formate as the primary products. We attribute this to hydrogen evolution at the working electrode, which alkalinizes the electrolyte. Under these conditions, C=C bond cleavage yields succinaldehyde; the nascent formaldehyde is then further oxidized to sodium formate.

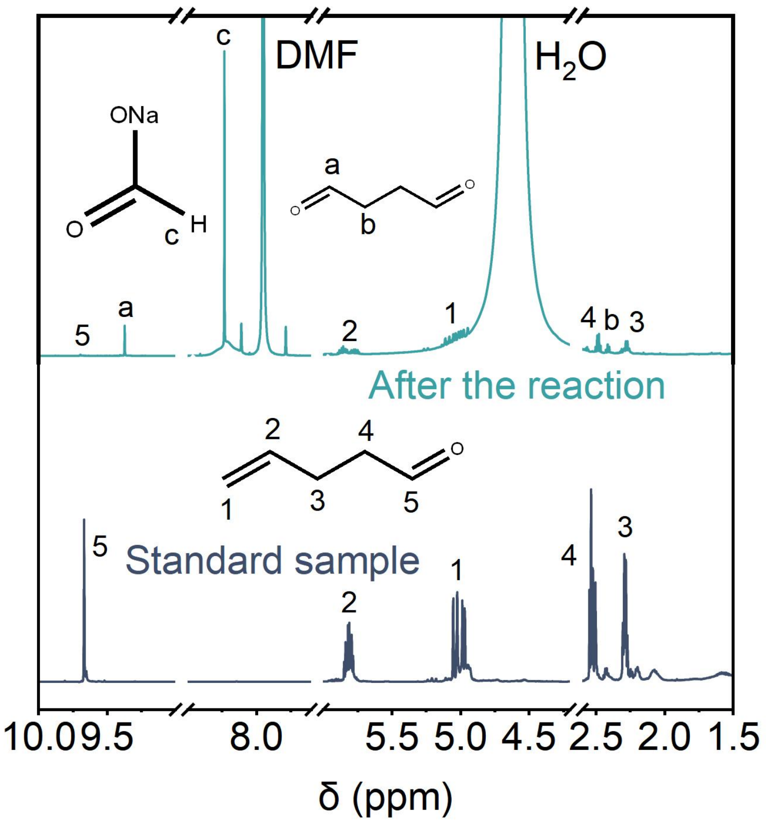

Figure S30 Comparison of the ^{1}HNMR spectrum of α-methylstyrene ketone before and after the reaction. α-Methylstyrene ketone exhibited only minor shifts in its characteristic NMR signals, with no new peaks detected. This behavior suggests that the direct linkage of carbonyl to the methyl group impedes the formation of the aldehyde-centered radical intermediate, thus suppressing reactivity.

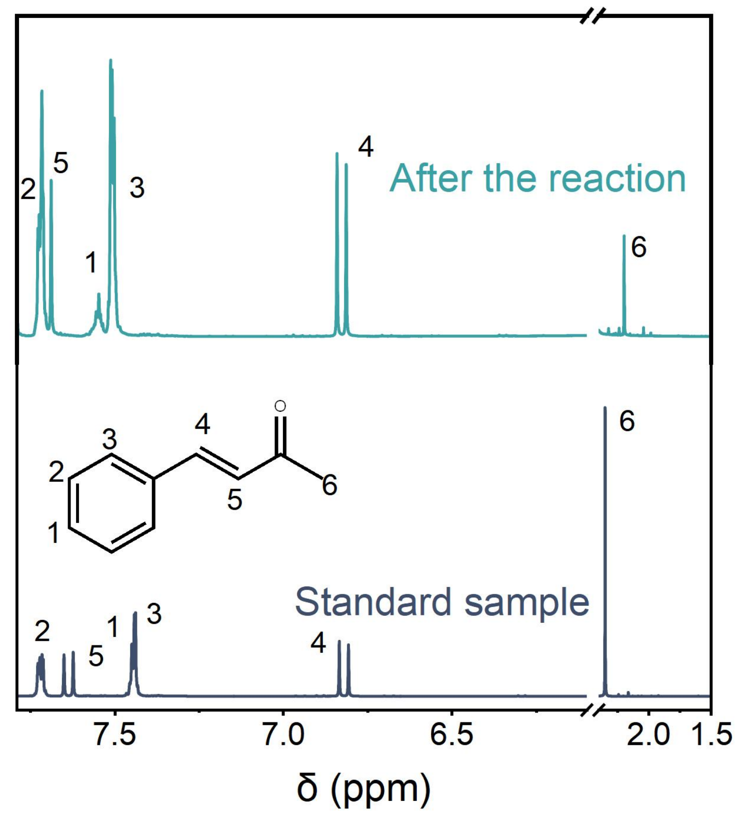

Figure S31 Comparison of the ^{1}HNMR spectrum of cinnamic acid before and after the reaction. Cinnamic acid showed the disappearance of the carboxylic proton resonance without new signals. Alkaline conversion to the sodium salt (–COO^{-}) alters the inductive effect on the C=C bond, preventing aldehyde-radical generation and, consequently, C=C oxidation.

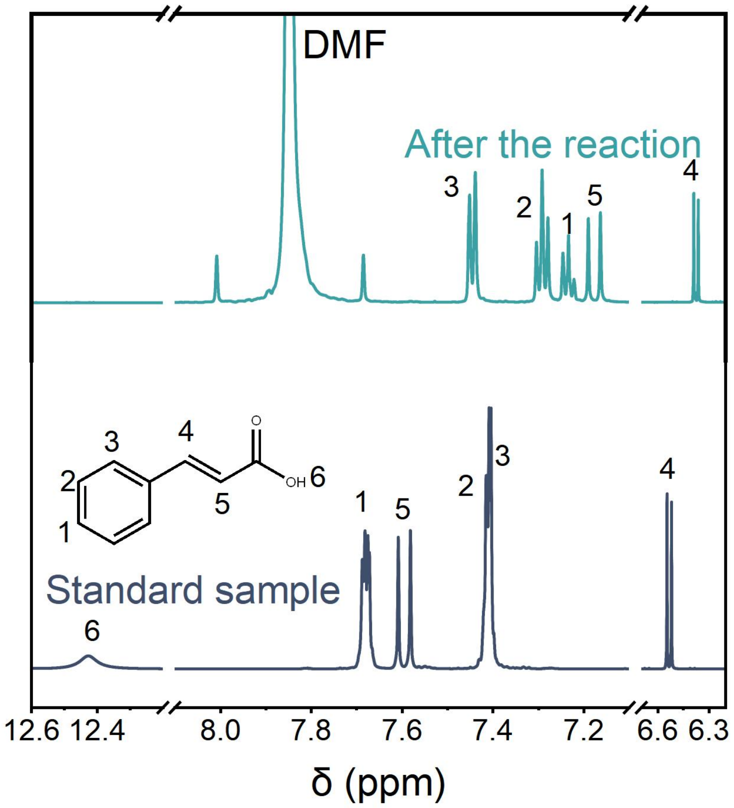

Figure S32 Comparison of the ^{1}HNMR spectrum of Citral before and after the reaction. Citral returned an NMR spectrum virtually identical to that of the starting material. This result reflects pronounced steric hindrance from its extended carbon chain, which markedly reduces double-bond reactivity.

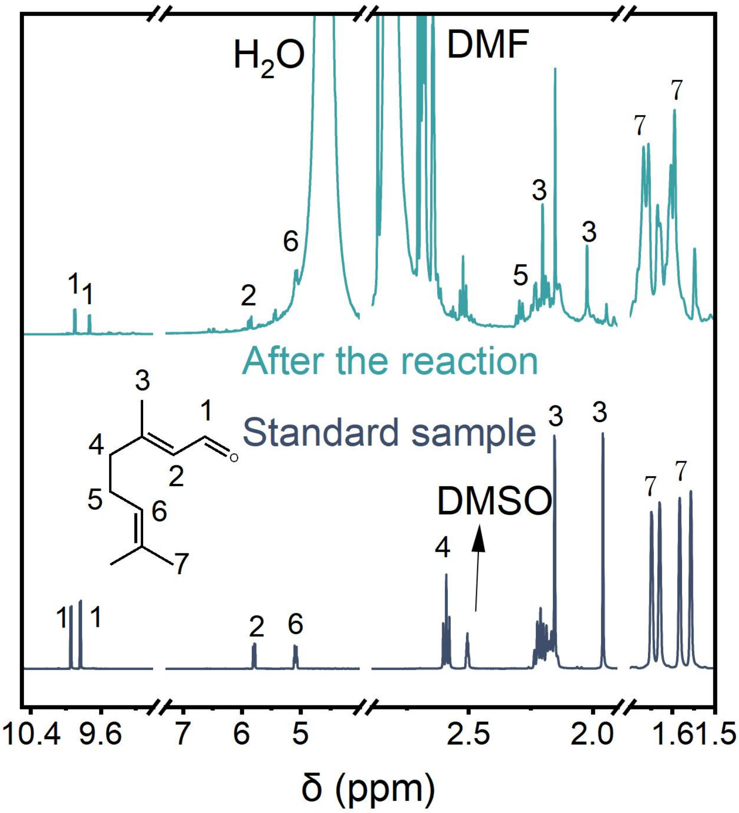

Figure S33 Comparison of the ^{1}HNMR spectrum of 2-butenal before and after the reaction. 2-butenal underwent C=C cleavage to give acetaldehyde; subsequent *OOH oxidation of acetaldehyde produced acetic acid.

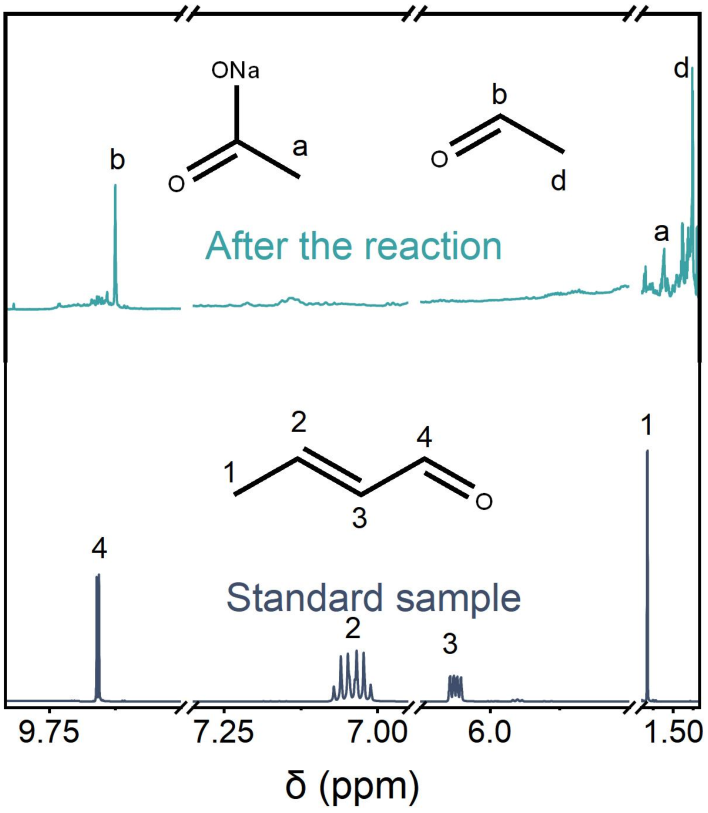

Figure S34 Comparison of carbon dioxide content between raw gas and post-reaction as measured by GC. This result suggested that glyoxal was converted to carbon dioxide during our reaction.

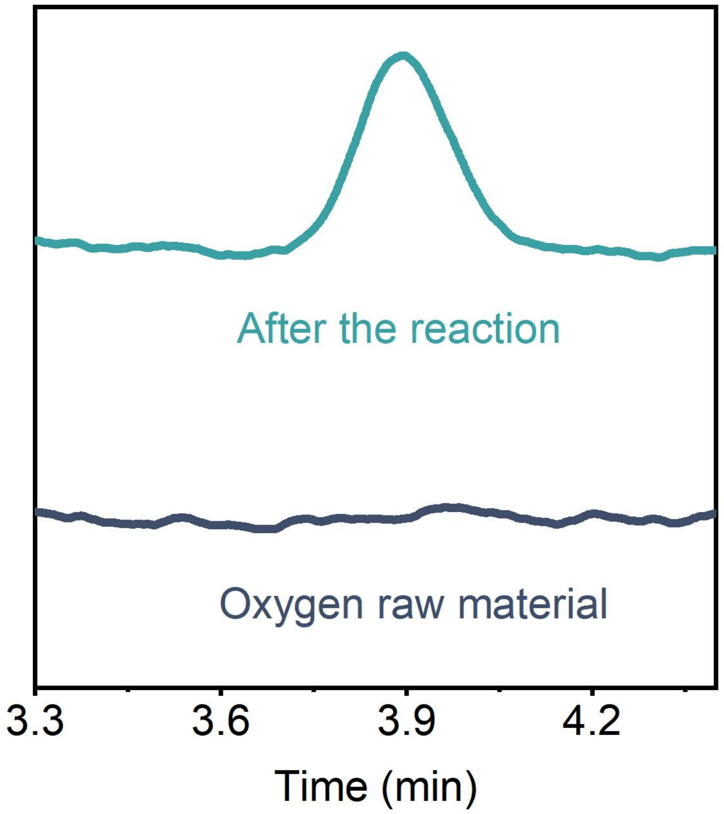

Table S1 Comparison of selectivity and yields of various organic substrates

Substrate	Product	Yields (%)	Selectivity (%)
4-pentenal	Formic acid	80	90.2
	Malondialdehyde	8.7	9.8
Benzalacetone	/	/	/
Cinnamic acid	/	/	/
Citral	/	/	/
3-butenal	Acetaldehyde	40	40.8
	Acetic acid	58	59
Cinnamaldehyde	Benzaldehyde	>99	>99
